# Paraptosis: a unique cell death mode for targeting cancer

**DOI:** 10.3389/fphar.2023.1159409

**Published:** 2023-06-15

**Authors:** Sweata Hanson, Aiswarya Dharan, Jinsha P. V., Sanjay Pal, Bipin G. Nair, Rekha Kar, Nandita Mishra

**Affiliations:** ^1^ School of Biotechnology, Amrita Vishwa Vidyapeetham, Kollam, Kerala, India; ^2^ Department of Cell Systems and Anatomy, UT Health San Antonio, San Antonio, TX, United States

**Keywords:** cancer, paraptosis, programmed cell death, alternative cell death, cancer therapy, apoptosis

## Abstract

Programmed cell death (PCD) is the universal process that maintains cellular homeostasis and regulates all living systems’ development, health and disease. Out of all, apoptosis is one of the major PCDs that was found to play a crucial role in many disease conditions, including cancer. The cancer cells acquire the ability to escape apoptotic cell death, thereby increasing their resistance towards current therapies. This issue has led to the need to search for alternate forms of programmed cell death mechanisms. Paraptosis is an alternative cell death pathway characterized by vacuolation and damage to the endoplasmic reticulum and mitochondria. Many natural compounds and metallic complexes have been reported to induce paraptosis in cancer cell lines. Since the morphological and biochemical features of paraptosis are much different from apoptosis and other alternate PCDs, it is crucial to understand the different modulators governing it. In this review, we have highlighted the factors that trigger paraptosis and the role of specific modulators in mediating this alternative cell death pathway. Recent findings include the role of paraptosis in inducing anti-tumour T-cell immunity and other immunogenic responses against cancer. A significant role played by paraptosis in cancer has also scaled its importance in knowing its mechanism. The study of paraptosis in xenograft mice, zebrafish model, 3D cultures, and novel paraptosis-based prognostic model for low-grade glioma patients have led to the broad aspect and its potential involvement in the field of cancer therapy. The co-occurrence of different modes of cell death with photodynamic therapy and other combinatorial treatments in the tumour microenvironment are also summarized here. Finally, the growth, challenges, and future perspectives of paraptosis research in cancer are discussed in this review. Understanding this unique PCD pathway would help to develop potential therapy and combat chemo-resistance in various cancer.

## 1 Introduction

Programmed cell death (PCD) is a universal event in various hosts, from microorganisms to higher eukaryotes. Programmed cell death is required for normal development and tissue homeostasis to maintain constant cell turnover (Tower, 2015). There are different classes of cell death machinery divided according to their morphological appearances involving apoptosis (Type I), autophagy (Type II) and cytoplasmic cell death (Type III) (Galluzzi et al., 2018). These pathways play a major role in regulating tumour immunity. Evidence has proven the therapeutic application of compounds targeting altered cell death pathways or the PCD network in various cancers (Ouyang et al., 2012). Numerous papers have listed the significance of PCD in different physiological processes. PCD removes damaged or toxic cells from our system to maintain cellular homeostasis. Immunity against various tumour cells is considerably dependent on PCD, either pro-tumour or anti-tumour, primarily based on intracellular molecules released during the process (Liu et al., 2022a). An increase in resistance to PCD during ageing is associated with reduced immune system activity; on the other hand, malignancy rate is higher with ageing (Tower, 2015). As apoptosis is considered the primary form of PCD, many reviews showcase the mechanism, modulators, pathways, and importance of apoptosis (Elmore, 2007; Xu et al., 2019). Other literature has also listed the comprehensive role of apoptosis in targeting cancerous cells (Baig et al., 2016; Pistritto et al., 2016; Carneiro and El-Deiry., 2020). A wide range of studies have shown that mutations in genes responsible for apoptosis during development has led to the discovery of alternative forms of cell death.

There has been extensive research focused on targeting the alternate cell death pathways, which showed their importance in controlling different metabolic processes and in the onset and progression of innumerable diseases (Li et al., 2020; Nah et al., 2020a; Qiu et al., 2022; Yan et al., 2022). The molecular mechanism behind natural compounds and small molecules induced alternative cell death pathways like necroptosis, ferroptosis, pyroptosis, cuproptosis, etc. have been reviewed and their role in tumour immunity has been discussed (Tong et al., 2022). Paraptosis is a vital cell death machinery in generating an anti-tumour effect in various cancer subtypes. Many natural and synthetic compounds have been identified to be potential targets for inducing paraptosis in different cancer cell lines (Fontana et al., 2020). Even though there have been a handful of reviews showcasing the importance of varying cell death pathways, there is no report of literature that briefly summarizes the critical cell death pathways and their mechanism. This review also stands out by giving a detailed explanation of all the modulators and signalling pathways of paraptosis, with a list of the recent development in the field, including the use of nanomedicines and metal complexes as paraptosis inducers, the potential application of paraptosis in combinatorial and photodynamic therapy (Wang et al., 2022), and existing *in vivo* studies in mouse xenograft model of cancer. Finally, the current research gaps and the potential prospects of paraptosis in cancer therapy have been discussed.

## 2 Apoptosis: the most studied cell death pathway

“Apoptosis” was among the first programmed cell death pathways extensively studied. Ker, Wyllie and Currie coined this term in 1972 by observing apoptotic cell death in a human tissue cell type with condensed and fragmented nuclei (Kerr et al., 1972). The first evidence that this cell death is an active process is from the study of genes in *Caenorhabditis elegans* that control apoptotic cell death (Hengartner, 1997). Genetic studies have revealed that the apoptotic cell death pathway is conserved through evolution from worms to mammals where ced-4, ced-3 and ced-9 in *C*. *elegans* are homologous to bcl-2, apaf-1 and caspase-9 in mammals. The early stage of apoptosis is marked by the shrinking of cells, condensation of chromatin (pyknosis), nuclear fragmentation (karyorrhexis) and plasma membrane blebbing, as evidenced by light microscopic studies. Other significant features of apoptosis include the lack of inflammatory reactions. Apoptotic cells are quickly phagocytosed, and anti-inflammatory cytokines or other cellular constituents are not released into the surrounding tissues. Caspases are the key enzymes which carry out the death process. Twelve genes have been discovered in the human genome that encodes 12 caspases, such as caspase-1 to caspase-10 and caspase-12, 14. Caspases are usually synthesized as inactive zymogens and categorized into two distinct classes: initiator (caspase 8, 9) and effector (caspase 3, 6, 7) caspases (Cohen, 1997; Li and Yuan, 2008) mediating cell death.

In contrast, caspases 1, 4, 5, 11, and 12 are proinflammatory as they regulate cytokine maturation during inflammation (Li and Yuan, 2008). Apoptosis can be triggered by various injurious stimuli (internal or external), such as heat, radiation, hypoxia, hormones (corticosteroids) and cytotoxic anticancer drugs that cause DNA damage (Elmore, 2007). Apoptosis can be executed through two pathways - intrinsic and extrinsic. The intrinsic pathway is mediated by mitochondrial proteins that sense various stress signals like radiation causing DNA damage, lack of growth factor, and toxins.

The extrinsic pathway is also known as the death receptor pathway of apoptosis (Igney and Krammer, 2002). It is triggered by the surveilling NK cells or macrophages that produce death ligands. The binding with death receptors (DRs) in the target cell membrane leads to the activation of procaspase 8 to caspase 8 (D’Arcy, 2019). Both intrinsic and extrinsic pathways culminate in the activation of executioner caspases. These caspases further activate endonucleases that degrade DNA; and proteases that degrade the nuclear and cytoskeletal proteins. This results in the collapse of the cell (Slee et al., 2001).

The principal treatment strategy against cancer is chemotherapy, but the major problem with cancerous cells is their ability to resist. This resistance can be of two major types: intrinsic and acquired resistance. In the case of intrinsic resistance, the resistance mediating factors will already be present in the tumour cells, but the onset of acquired resistance will be during treatment (Holohan et al., 2013). This problem of apoptosis resistance has led to the urgent need to investigate different alternate forms of cell death pathways such as anoikis, autosis, cornification, endosis, ferroptosis, methuosis, mitotic catastrophe, necroptosis, NETosis, parthanatos, pyroptosis and paraptosis.

## 3 Alternative cell death pathways

**FIGURE 1:**
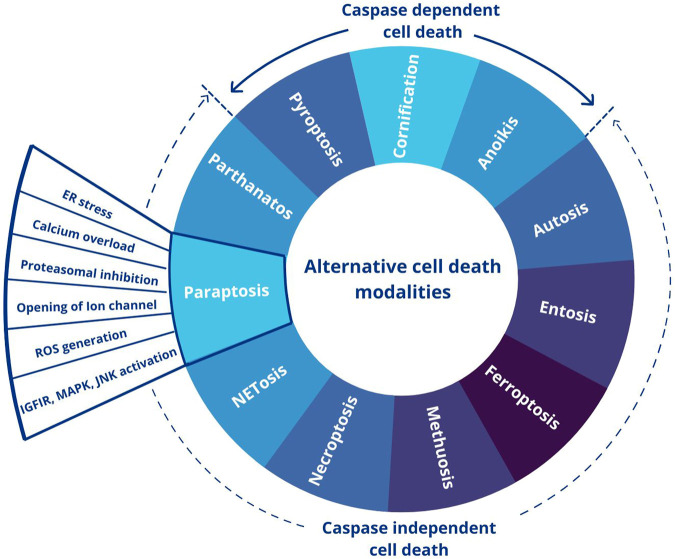
Different forms of alternative cell death pathways. This figure was generated using Canva (www.canva.com).

Even though apoptosis is regarded as the primary cell death pathway, the understanding of the significance of alternate forms of cell death was initiated by the study in mice with a mutation in genes responsible for canonical apoptosis mediators (Yuan and Kroemer, 2010). The presence of an alternate form of cell death is shown by morphological observations in the normal development of mammalian embryos (Schweichel and Merker, 1973). Alternate cell death pathways can mainly be of two types: caspase-independent and caspase-dependent. The former is a death that occurs by a signal that usually induces apoptosis but fails to initiate caspase activation (Tait and Green, 2008). The classification of the alternate cell death pathways based on their dependence on caspase activation is illustrated in Figure 1, highlighting paraptosis, a caspase-independent PCD.

### 3.1 Anoikis

Cells in tissues require a specific extracellular matrix and cell-to-cell attachment. Anoikis, discovered in early 1990, is an apoptosis-like cell death induced due to cell detachment from the ECM (Gilmore, 2005). These resistant cells metastasize in the body by becoming anchorage-independent and undergo epithelial-to-mesenchymal transition leading to dysregulated metabolism in cancer cells (Paoli et al., 2013). Lack of integrin signalling, downregulation of EGFR expression, inhibition of extracellular receptor kinase 1 (ERK1) signalling, and overexpression of Bim, a Bcl-2 family protein, are some of the features of anoikis. Anoikis can be induced by the intrinsic or extrinsic pathway (Taddei et al., 2012). Moreover, it was reported in neuroblastoma and lung cancer cells (A549) caspase-8 acts as an initiator caspase responsible for promoting anoikis (Bozzo et al., 2006; Chiou et al., 2012; Sattari Fard et al., 2023).

### 3.2 Cornification

Cornification is a unique form of programmed cell death in epidermal keratinocytes. The keratinocytes convert into “corneocytes”, forming the outermost skin barriers such as nails and hair (Eckhart et al., 2013; Jaeger et al., 2019). Proteins like loricrin, involucrin, filaggrin (a cysteine-rich protein), ‘small proline rich’ proteins (SPRRs) and keratin are found to be present on the thick and protective epidermal layer. These epidermis-specific proteins and lipids (e.g., fatty acids and ceramides) provide structural stability, mechanical resistance, elasticity, and hydration to the skin (Gutowska-Owsiak et al., 2018). Transglutaminase enzyme helps catalyse the cross-linking between the structural proteins to form the protein part of the cornified envelope (Candi et al., 2005). Caspase-14 plays a vital role in the cornification process (Denecker et al., 2007).

### 3.3 Pyroptosis

The term pyroptosis is derived from the Greek roots “pyro”, which refers to fire or fever, and “ptosis”, which suggests falling (Cookson and Brennan, 2001). It is an anti-inflammatory form of programmed cell death induced by caspase-1/4/5/11 and some inflammasomes. This cell death pathway includes cell swelling, plasma membrane lysis, chromatin fragmentation and release of intracellular proinflammatory contents (Fang et al., 2020). Chromatin condensation and DNA fragmentation occur during pyroptosis, but it differs morphologically from apoptosis by having an intact nucleus (Jorgensen and Miao, 2015). During pyroptosis, a pattern recognition receptor containing inflammasome identifies specific pathogen-associated molecular patterns (Shi et al., 2017). This recognition leads to a large supramolecular assembly of ASC (inflammasome adaptor protein), which links the nucleotide-binding domain and leucine-rich repeat-containing receptors to caspase-1. This complex induces apoptosis through canonical or noncanonical inflammasome pathways (Xu et al., 2018). Pyroptosis has been reported to serve an essential role in the treatment of cancer (Xia et al., 2019; Huang et al., 2022b).

### 3.4 Autosis

Autosis is an autophagy-dependent, non-apoptotic form of cell death characterized by the ballooning of the perinuclear space (PNS), initiated by the excessive accumulation of the autophagosomes (Nah et al., 2020a). Autophagy-inducing peptides, starvation, and hypoxia conditions can induce autosis. Very mild chromatin condensation and the dependence on Na^+^/K^+^-ATPase, an upstream regulator of other cell death pathways, are essential in highlighting their unique role in cell death (Liu and Levine, 2015).

### 3.5 Entosis

Overholtzer et al. (2007) described “entosis”, a new mode of non-apoptotic cell death pathway in mammary epithelial cells. Entosis/cell cannibalism is a process like anoikis triggered by the detachment of mammary epithelial cells from the ECM, followed by the invasion of one cell into the other. This invasion requires adherens junctions and occurs in the absence of integrin signalling. Most entotic cells die after they are internalized (Krishna and Overholtzer, 2016). It requires Rho GTPase and activation of the downstream effector, ROCK, in internalizing cells. Entosis is insensitive to the inhibition of Bcl-2, requires myosin/actin cytoskeleton and undergoes lysosome-mediated degradation that is autophagy-independent (Liu et al., 2019). Kras, Rac, and tumour suppressors like E-cadherin induce entosis (Sun et al., 2014).

### 3.6 Ferroptosis

Ferroptosis is a non-apoptotic cell death pathway induced by intracellular iron and iron-dependent lipid peroxidation (Sun et al., 2020). The term ferroptosis was coined by Stockwell et al., 2012 while studying the cell death mechanism induced by erastin. Rounding up of the cells with altered morphology in mitochondria and cristae structure marks its characteristic features. Molecules like erastin, RSL3, FIN56 etc., can initiate this cell death mechanism (Xie et al., 2016). Compared to apoptosis, ferroptosis is more immunogenic. Ferrostatin-1 is an inhibitor of ferroptosis (Dixon et al., 2012; Sun et al., 2020). Iron chelators and antioxidants can also inhibit ferroptosis by interfering with intracellular iron. Inactivation of GPX4 (Glutathione peroxidase 4) and depletion of GSH (Lu et al., 2017; Lei et al., 2022) trigger ferroptosis (Lu et al., 2017).

**FIGURE 2:**
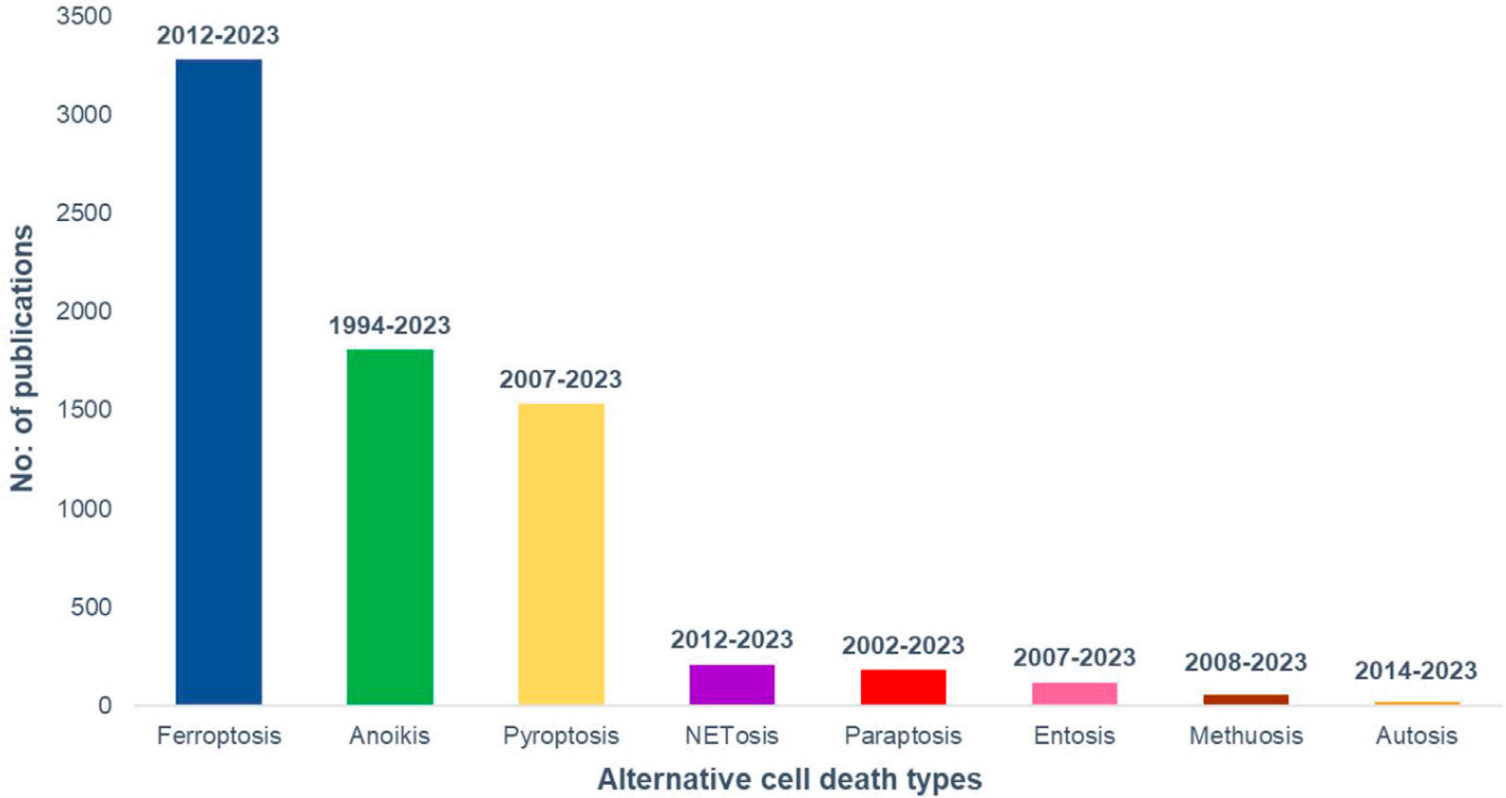
Research growth in different modes of alternative cell death in cancer till January, 2023. PubMed data taken for the analysis.

The number of research publications and the study duration on cancer and different alternative programmed cell death pathways are taken from PubMed and plotted in Figure 2 to understand the current status of the research in the field.

### 3.7 Methuosis

Methuosis is a caspase-independent form of cell death caused by the accumulation of fluid-filled cytoplasmic vacuoles followed by cell detachment. It was first discovered in glioblastoma cells, induced by increased expression of Ras GTPase (Chi et al., 1999). These are single membrane-bound vacuoles derived from macropinosomes with markers like GTPase Rab7 (late endosomes) and membrane glycoprotein Lamp-1 (lysosome) (Nirmala and Lopus, 2020). H-RAS (G12V) and K-RAS4B (G12V) can activate GTPase Rac1, thereby controlling the formation of macropinosomes. The lack of hydrolytic enzymes and the non-acidic nature of these vacuoles categorize them as non-functional late endosomes (Maltese and Overmeyer, 2014).

### 3.8 Mitotic catastrophe

A mitotic catastrophe is a form of cell death in higher eukaryotes (Vitale et al., 2011). This type of cell death induced by abnormal mitosis is related to voluntary premature chromosome condensation and the formation of large cells with multiple micronuclei (Ianzini and Mackey, 1997). The morphological features associated with these pathways resemble those during a failed mitosis, including chromosome breaks and karyokinesis deficiency leading to gross nuclear alteration (micro nucleation and multinucleation). This cell death pathway can affect the integrity of the genetic material, and some inducers trigger uncharacterized pathways (hypothermia and heat shock responses) (Sekhar et al., 2007).

### 3.9 Necroptosis

Necroptosis is regarded as the programmed form of necrotic cell death, which shows a similar mechanism and morphology to apoptosis and necrosis, respectively (Christofferson and Yuan, 2010). It is a caspase-independent cell death pathway, which is mediated by Receptor-Interacting Protein 1 (RIP1), RIP3, and Mixed Lineage Kinase Domain-Like (MLKL) proteins (Gong et al., 2019). They are inhibited by necrostatin-1 (Nec-1) by blocking the RIPK1 activity (Degterev et al., 2008). A range of signals, including the tumour necrosis factor receptors (TNFR), pattern recognition receptors (PRRs), T-cell receptors (TCRs) (Lalaoui et al., 2015) various chemotherapeutic drugs, and environmental stresses such as hypoxia (Huang et al., 2013) can activate necroptotic cell death pathway. Necroptosis plays a significant role in controlling viral infection and regulating cancer, including oncogenesis, cancer metastasis, cancer immunity, and cancer subtypes (Stoll et al., 2017; Seehawer et al., 2018).

### 3.10 NETosis

Neutrophils being the major component of innate immunity, act by phagocytizing the pathogens by secreting granules that are enriched in cytotoxic enzymes or by expelling neutrophil extracellular trap (NET) that are rich in DNA, modified histones and cytotoxic enzymes (Thiam et al., 2020). The cells that undergo NETosis are marked by cytoplasmic vacuolization, spreading of cells, change in cell shape, chromatin condensation and breakdown of the nuclear membrane. NETosis is insensitive to caspase inhibition (Galluzzi et al., 2012). Ligands for receptors like the G protein-coupled receptor (Gupta et al., 2014) can initiate NETosis.

### 3.11 Parthanotos

The term Parthanatos, is a portmanteau term (parthanatos) derived from “par” (for PAR polymer, synthesized following PARP-1 activation), and “Thanatos,” the personification of death in Greek mythology (Andrabi et al., 2008; David et al., 2009; Wang et al., 2009). Their molecular mechanisms (suicide hypothesis) include induction and overexpression of PARP-1, accumulation of PAR polymer, mitochondrial depolarization, nuclear AIF translocation, unrestrained exhaustion of cytosolic and nuclear NAD + resulting in cells committing suicide (Yu et al., 2003; Andrabi et al., 2006). PAR interacts with different proteins and this interaction modulates their physiological function (Gagné et al., 2008; Krietsch et al., 2012). Parthanatos are involved in the pathogenesis of neurodegenerative disorders, cerebrovascular diseases, spinal cord injury, and glioma (Wang and Ge, 2020).

Different forms of programmed cell death occur depending on various cell types, types and duration of stimuli, and specific cellular and molecular changes induced by the stimuli. During cell death, the cells undergo morphological, cellular, and biochemical changes. While nuclear condensation, DNA fragmentation, caspase activation and apoptotic body formation distinguish apoptosis; changes like cytoplasmic vacuolation, ER stress, organellar dysfunction and MAPK activation define paraptosis as an alternate PCD. Comparing the features of paraptosis with other cell death modalities will give a clear picture of the differences that make them unique, as shown in Table 1.

**TABLE 1:** Features of paraptosis compared with other major forms of cell death.

Sl. No:	Type of cell death	Nuclear changes	Cell membrane	Cytoplasmic changes and vacuolation	Organellar changes	Mechanism of action/Biochemical changes
1	Apoptosis Nah et al. (2020a)	Nuclear fragmentation and chromatin condensation	Plasma membrane intact, altered lipid orientation	No vacuoles	Cell shrinkage	Caspase activation
					Release of cytochrome c, mitochondrial dysfunction	PARP cleavage, phosphatidylserine exposure
2	Anoikis Taddei et al. (2012); Sattari Fard et al. (2023)	DNA damage	Detachment from ECM	**_**	Cytoskeletal and metabolic changes, disruption of mitochondria	Caspase-8 activation, autophagy induction, ROS increase
3	Cornification Denecker et al. (2007); Kroemer et al. (2009); Eckhart et al. (2013); Murata et al. (2018)	DNA degradation	Modification of plasma membrane	Extrusion of lipids in the extracellular space	Increase in lysosome-like bodies; the disappearance of mitochondria, Golgi apparatus, ribosomes, and ER; cornified envelope formation	Calcium-mediated
						Caspase-14 activation
4	Pyroptosis Cookson and Brennan. (2001); Fang et al. (2020); Zhang et al. (2022)	DNA fragmentation, chromatin condensation, nucleus intact	Plasma membrane lysis	Cytoplasmic swelling	Cell swelling and release of intracellular pro-inflammatory contents	Caspase 1/4/5/11-activation, Gasdermin D cleavage
5	Autosis Nah et al. (2020b); Nah et al. (2020a)	Mild chromatin condensation, nuclear concavity	Focal plasma membrane rupture	Excessive accumulation of autophagosomes	Swollen mitochondria (ballooning of perinuclear space), fragmented ER, depolarization of mitochondrial membrane potential	Activation of autophagy, Rubicon-mediated
6	Entosis Overholtzer et al. (2007); Garanina et al. (2017); Kianfar et al. (2022)	Distorted nucleus	Actin build-up blebbing	Strong acidification of entotic vacuole	Cytoskeletal changes, relocation of organelles like Golgi complex	Mediated by E-cadherin and P-cadherin; Rho and ROCK activity
7	Ferroptosis Dixon et al. (2012); Capelletti et al. (2020); Li et al. (2020)	Normal nucleus	No rupture of plasma membrane; no membrane blebbing	No cytoplasmic swelling	Mitochondria shrinkage with ruptured outer membrane	Lipid peroxidation due to iron-dependent depletion of GSH and Glutathione peroxidase inactivation; MAPK activation; lipid ROS accumulation; ATP depletion
8	Methuosis (Maltese and Overmeyer, 2014)	DNA fragmentation varies, no chromatin condensation	Rupture of the plasma membrane, no membrane blebbing	Cytoplasmic vacuolation, Vacuoles in macropinosomes and endosomes	Cell swelling	Caspase activation
						ATP depletion
9	Mitotic Catastrophe Kimura et al. (2013); Sazonova et al. (2021)	DNA damage, multinucleation and/or micronucleation	_	_	Permeabilization of mitochondrial networks and release of cytochrome c; depletion of centrosomal proteins	Caspase-2 activation, abnormal increase in cyclinB1 level, mitotic arrest, mitochondrial release of pro-apoptotic proteins
10	Necroptosis Chen et al. (2016); Gong et al. (2019)	No nuclear fragmentation	Plasma membrane swelling and rupture	Cytoplasm swelling and vacuolization	Mitochondrial and organelle swelling, mitochondrial and lysosomal membrane permeability affected	ATP depletion
						ROS production
						Calcium and sodium influx; RIPK and MLKL activation
11	NETosis Fuchs et al. (2007); Thiam et al. (2020)	Decondensed chromatin, destruction of the nuclear envelope	Pores in the plasma membrane	Cytoplasmic vacuoles	Cytoskeletal disassembly, remodelling of membranous organelles like ER and mitochondria	Caspase-independent
						Induced by ROS production; GPCR activation; formation of NETs; NADPH oxidase activity by mitochondria
12	Parthanatos Andrabi et al. (2008); Maltese and Overmeyer, (2014); Huang et al. (2022a)	Chromatin condensation, DNA fragmentation	No rupture in the plasma membrane, and no membrane blebbing	No visible vacuoles	No cell swelling, mitochondrial membrane permeability affected	Caspase activation
						ATP depletion
						PARP-1 overexpression
13	Paraptosis Kim et al. (2021); Li et al. (2022b); Sperandio et al. (2000)	DNA fragmentation absent	**_**	Extensive cytoplasmic vacuolation	Swelling of ER or/and mitochondria	Affecting mitochondrial membrane permeability,
						ATP depletion, Ca2+ imbalance, opening of K+ ion channels
						MAPK activation, disruption of sulfhydryl homeostasis

### 3.12 Paraptosis

Sperandio et al. (2000) coined the term “paraptosis”. They reported the role of Insulin growth factor receptor 1 (IGFIR) in inducing programmed cell death in 293T cells and mouse fibroblast cells (Sperandio et al., 2000). This cell death differed from apoptosis due to the lack of nuclear fragmentation, formation of apoptotic bodies, chromatin condensation, and caspase-induced cell death. The paraptotic cells showed extensive cytoplasmic vacuolation with the dilation of ER/mitochondria. Vacuolation mode of cell death has been reported previously in neural development, where it was described as non-lysosomal disintegration (Type 3A) or cytoplasmic type (Type 3B) (Yuan et al., 2003). Paraptosis was categorized under type 3B due to its morphological evidence with the lack of autophagic vacuoles (Ghosh et al., 2016). Caspase and autophagy inhibitors did not affect this mode of cell death. Cycloheximide treatment stopped cytoplasmic vacuolation-mediated cell death, indicating that protein synthesis is required for mediating paraptosis. Paraptosis is a unique cell death pathway with a broader role during development, neurodegeneration, viral and bacterial infection, Glaucoma, etc. Paraptosis has now gained attention from different studies involving natural compounds (Lee et al., 2016), nanomedicines (Zhou et al., 2018; Zheng et al., 2021), monoclonal antibodies (Rojpibulstit et al., 2014), combinatorial therapies, etc. Metallic death was observed in dying neurons. Metal complexes like copper complex (Gandin et al., 2012; Chen et al., 2017), radiation therapies (Hu et al., 2016; Wang et al., 2020), and photodynamic therapy (Kessel, 2019; Kessel, 2020) have also shown to induce paraptosis.

**FIGURE 3:**
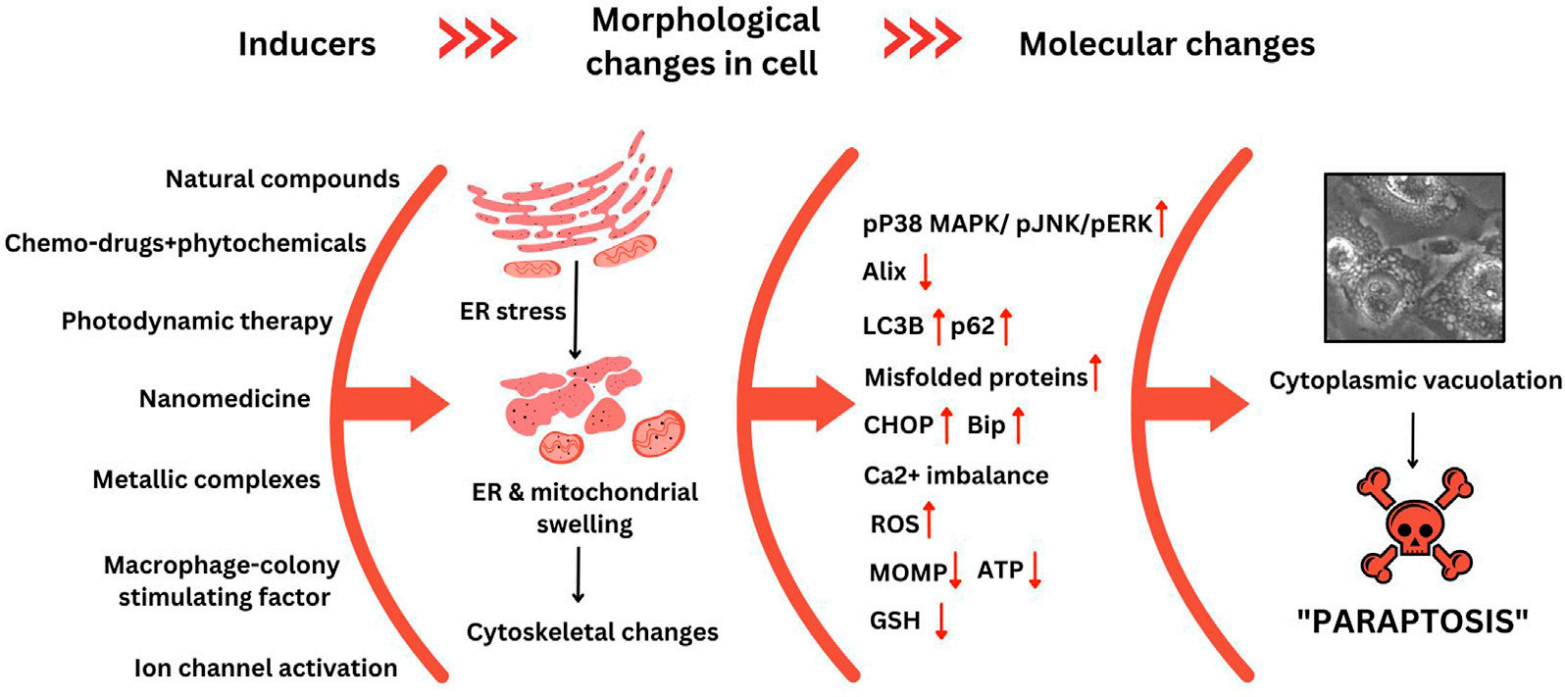
Cellular and molecular changes observed during paraptosis. This figure was generated using Canva (www.canva.com) and data from Binoy et al., 2019.

Different inducers triggering paraptosis, the changes in cellular morphology, and other molecular markers in cancer cells have been outlined in Figure 3. It is essential to understand the molecular mechanism of how and why paraptosis happens and the various signal transduction pathways involved.

## 4 Mechanism of paraptosis

Paraptosis is a caspase-independent programmed cell death pathway. Interestingly, Sperandio et al., during their initial studies, reported the activation of the MAP kinase pathway essential for paraptosis-mediated cell death. Later, listed AIP1/Alix (human homolog of BRO1) was found to inhibit paraptosis through inhibition of MAPK activity (Sperandio et al., 2004). This pathway is characterized by extensive cytoplasmic vacuolation that involves swelling of ER/mitochondria. A detailed understanding of how and why paraptosis happens and the various signal transduction pathways involved is getting gradually uncovered. Paraptosis has been reported to be induced through several mechanisms, including the expression of Insulin Growth Factor 1 Receptor (IGF1R), proteasome inhibition and ER stress, Reactive oxygen species (ROS) generation, Ca^2+^ influx into mitochondria or opening of the ion channel etc.

### 4.1 Insulin growth factor 1 receptor (IGF1R) induced paraptosis

**FIGURE 4:**
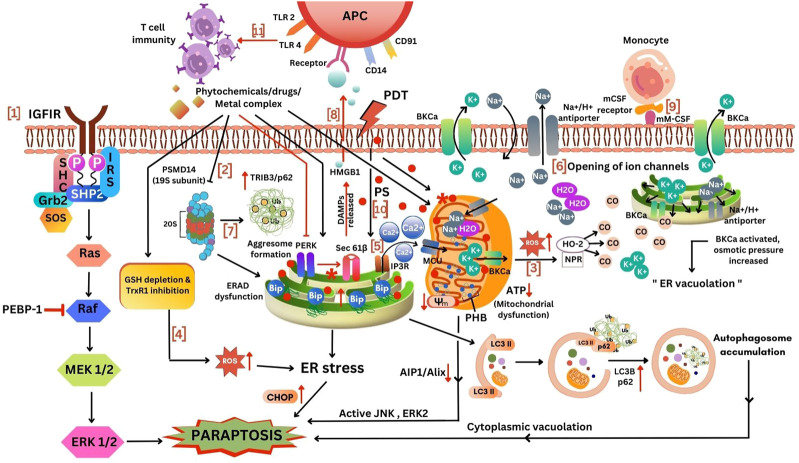
Different mechanism for induction of paraptosis. 1, IGFIR induced paraptosis via MAPK activation; 2, Proteasomal and ERAD inhibition mediated ER stress; 3, Increased ROS production from mitochondria to cytoplasm activated HO-2 and NPR to release CO thereby opening BKCa channel leading to paraptosis; 4, ROS increase due to the inhibition of TrxR1 and GSH depletion inducing ER stress mediated paraptosis; 5, Ca^2+^ influx via MCU triggers mitochondria mediated paraptosis; 6, Opening of ion channels, low K^+^ ions inside ER and mitochondria allows the transport of Na^+^ and H_2_O into the cell thereby causing swelling of ER and mitochondria; 7, ERAD dysfunction due to the inhibition of proteasome leads to aggresome formation inducing ER stress mediated paraptosis; 8, Stimulation of immune response; 9, mCSF expressing cancer cells recognized by monocytes via activation of BKCa channel induced paraptosis; 10, The effect of photosensitizers in ER and mitochondrial inducing paraptosis; 11, Deletion of PERK induces sec61β-linked paraptosis leading to the release of damage-associated molecular patterns (DAMPs) such as HMGB1 thereby promoting monocytic-lineage inflammatory dendritic cells enhancing T-cell immunity. This figure was generated using Canva (www.canva.com).

Sperandio et al. (2004) reported that the onset of cell death requires the expression of the Insulin Growth Factor 1 Receptor and membrane localization of the intracytoplasmic domain IGFIR-IC. Though this was classified under the dependence receptor category, the presence of the ligand was found to be essential. The group conducted site-directed mutagenesis studies, which suggested that paraptosis requires an intact kinase domain on the membrane-bound IGF-IR-IC. This finding prompted them to study the involvement of the mitogen-activated protein kinase (MAPK) family of proteins. The assumption was proved right using pathway-specific inhibitors and RNAi-mediated studies, in which MAPK/ERK and JNK were found to be activated during paraptosis. Since caspase-9 is triggered due to MAPK kinase signalling, the group studied the activation of caspase-9 upon paraptosis induction. They postulated that the molecular switch that deviates the cell death pathway towards paraptosis was achieved when ERK-2 phosphorylates caspase-9 at Thr125, thus inhibiting apoptosis. Even though caspase-9 has a role in apoptosis and paraptosis, paraptosis does not require the processing of caspase-9 by the zymogens. Literature has shown the link between BRO1 (yeast) to the MAPK pathway. Their studies reported that the overexpression of AIP1 can inhibit the phosphorylation of MAPK and the cell death induced by IGF-IR-IC, explaining the inhibitory role of AIP1/Alix as the endogenous inhibitor of paraptosis as shown in Figure 4.

The role of MAPK signalling also led to the elucidation of the role of PEBP in paraptosis. The IGF-IR-IC induced cell death was reduced by the PEBP/Raf kinase inhibitor protein (RKIP), which is a well-known Raf kinase inhibitor (shown in Figure 4) and apoptosis inhibitor through JNK phosphorylation but not MAPK phosphorylation (Sperandio et al., 2010).

Other than IGFIR receptor, activation of potassium ion channels and receptors like vanilloid receptor subtype 1 (VR1) (Jambrina et al., 2003), TAJ/TROY (Wang, 2004), neurokinin-1 receptors (NK1R) (Castro-Obregón et al., 2002), EGF (Fombonne et al., 2004), 1-nitropyrene (1-NP) (Asare et al., 2008), etc., have been reported for inducing paraptosis-like cell death. More recently, the role of the c-Jun N-terminal Kinase (JNK) and mitogen-activated protein kinase (MAPK) family of proteins were studied through different ways, including RNAi and CRISPR/Cas9 gene editing techniques, respectively. SHP2, an upstream intermediary of the MAPK pathway shown in Figure 4, has been identified as a critical target of paraptosis using CRISPR/Cas9 technique (Li et al., 2022a). Another study indicated that the binding of the peptide neurotransmitter substance P to its receptor, neurokinin-1, also induced a non-apoptotic form of death similar to paraptosis (Castro-Obregón et al., 2002). ^125^I radiation induced paraptosis in HCT116 cells by activating the PI3K/AKT signalling pathway (Hu et al., 2016).

### 4.2 Proteasomal inhibition/ER stress-mediated paraptosis

Paraptosis being a programmed cell death, requires new protein synthesis. The inhibition of paraptosis when treated with cycloheximide/actinomycin D suggested that translation and transcription are necessary for paraptosis induction (Sperandio et al., 2000; Sperandio et al., 2004). The requirement for protein synthesis brings our attention to the endoplasmic reticulum (ER). ER, functions as the main centre for protein synthesis and sorting. Several ER-localized proteins help to render this function. Studies have reported that the accumulation of newly synthesized misfolded proteins in the ER leads to ER stress and unfolded protein response (UPR) which can be due to proteasomal inhibition. Many natural compounds and metallic complexes listed in Table 2 have been shown to induce proteasomal inhibition by inhibiting one or more proteases in the 20S core of the 26S proteasome. The three proteases are trypsin-like, chymotrypsin-like and caspase-like proteases. The misfolded proteins in the cells are targeted for removal through the Ubiquitin Proteasomal System (UPS) after being marked by a polyubiquitin chain. Therefore, the inhibition of the proteasome can lead to the accumulation of polyubiquitinated proteins inside the cell. ER is connected to mitochondria via mitochondria-associated membranes (MAMs) that have a significant role in Ca^2+^ homeostasis. Disturbance in ER homeostasis can be due to stress factors like hypoxia, pH change, starvation, Ca^2+^ imbalance, ATP depletion, etc. (Limonta et al., 2019). The ER stress can lead to Unfolded Protein Response (UPR) activation to balance homeostasis by increased synthesis of ER chaperone protein BiP (GRP78) for protein folding, decreased translation, and upregulation of C/EBP Homologous Protein (CHOP) (Binoy et al., 2019) (in Figure 4). The major proteins which are induced during ER stress are the Binding of immunoglobulin protein (BiP) and CCAAT/enhancer-binding protein homologous protein (CHOP) (Kroeger et al., 2012). BiP, also known as glucose-regulated protein 78 kD (Grp78) or heat shock 70 kDa protein. This protein encodes ER-localized HSP70-class chaperone involved in the folding of protein by binding to the misfolded ER proteins for further quality control (Mayer and Bukau, 2005). Cells rapidly upregulate *BiP* mRNA levels when confronted with ER stress (Kozutsumi et al., 1988). The chop, also known as Growth arrest and DNA damage gene 153 (Gadd153) and DNA damage-inducible transcript 3 (Ddit3), encodes a transcription factor that promotes apoptosis in response to uncontrolled ER stress (Zinszner et al., 1998). CHOP (C/EBP homologous protein) upregulation due to ER stress can happen during apoptosis and paraptosis and in other types of cell death. An alternate death pathway is activated in the cell only when its apoptosis machinery is non-functional (Chipuk and Green, 2005; Elmore, 2007; Tait et al., 2014). The genes and proteins that participate in programmed cell death can be the same but might result in different outcomes depending upon the cell, type of stimuli and its molecular targets. CHOP cannot be taken as the key protein for paraptosis. A comprehensive analysis of several markers and assays would be necessary to understand which pathway is getting activated during CHOP activation. This accumulation of misfolded proteins causes ER dilation, one of the typical morphological features during paraptosis. Many natural compounds like curcumin (Huang et al., 2017), 6-shogaol (Nedungadi et al., 2018), gambogic acid (Seo et al., 2019), manumycin A (Singha et al., 2013), etc., have been shown to induce its anticancer effect by binding to sulfhydryl groups of cysteine residues in newly synthesized proteins in the cancer cells. The presence of α, β-unsaturated carbonyl group in the compound forms an adduct through the Michael addition reaction leading to the misfolding of the newly synthesized proteins, which accumulated in the cytosol and the ER. Several studies have reported the elevated expression of ER stress markers during paraptosis (Binoy et al., 2019). The HSP 70 inhibitor, VER155008 induced paraptosis in anaplastic thyroid carcinoma cells was associated with the formation of cytoplasmic vacuoles, dilation of ER and increased mRNA levels of Bip and CHOP (Kim et al., 2014). The compound 6-shogaol has shown elevated accumulation of ubiquitinated proteins and ER stress during paraptosis by decreasing the chymotrypsin activity of the β5 subunit of the 26S proteasome subunit (Nedungadi et al., 2018), and plumbagin showed disruption of sulfhydryl homeostasis (Binoy et al., 2019). Similarly, dual inhibition of thioredoxin reductase (TrxR), an enzyme that controls redox, plays a vital role during proteasome-induced paraptosis (Seo et al., 2023). Disrupted heme export in endothelial cells can induce paraptosis via ER stress (Petrillo et al., 2018). A higher concentration of taxol-induced paraptosis in ASTC-a-1 cells was associated with the induction of cytoplasmic vacuolation (Chen et al., 2008). The cytoplasmic vacuoles formed during ER stress-induced paraptosis were found to be of ER origin, as the ER-resident protein calreticulin marked them. Paraptosis can be induced in MDA-MB-435S breast cancer cell lines by inhibiting the 19S subunit of the regulatory particle of 26S proteasome (PSMD14) (Lee et al., 2022), as shown in Figure 4. The RNA-seq data of paraptosis strongly supports the expression of the proteins, as mentioned earlier, through its differentially expressed genes (Pyrczak-Felczykowska et al., 2022).

**TABLE 2:** Paraptosis modulators.

Sl. No:	Modulator protein	Activators/Inhibitors	Role/function	Primary References
1	MAPK/ERK/JNK	Activates paraptosis	Mitogen-activated protein kinases have a role in both cell proliferation and cell death	Sperandio et al. (2000)
2	SHP2	Activates paraptosis upstream to MAPK	SHP-2, a cytoplasmic SH2 domain-containing protein tyrosine phosphatase, is involved in the signalling pathways of various growth factors and cytokines	Li et al. (2022a)
3	PEBP-1	Inhibits paraptosis	Raf kinase inhibitor	Sperandio et al. (2010)
4	Bip	Inhibits by activating UPR	BiP, also known as glucose-regulated protein 78kD(Grp78) and heat shock 70 kDa protein 5. HKH40A downregulates GRP78/BiP expression in cancer cells	Kim et al. (2014)
5	CHOP	It promotes paraptosis in response to uncontrolled ER stress	C/EBP(CCAAT/enhancer-binding protein) homologous protein (CHOP) is a pro-apoptotic transcription factor encoded by the DDIT3 gene. Also known as Growth arrest and DNA damage gene 153 (Gadd153)	Yoon et al. (2014a)
6	PERK	Inhibits paraptosis by activating UPR	Pancreatic ER kinase (PKR)-like ER kinase (PERK), is a UPR mediator membrane protein in the ER.	Mandula et al. (2022)
7	LC3B	Activates paraptosis	A well-known autophagy marker is the LC3-II (Microtubule-associated protein light chain 3B). They are generated because of the conjugation of cytosolic LC3-I to phosphatidylethanolamine that is present on the surface of autophagosomes	Kar et al. (2009)
8	Alix	Inhibits paraptosis	ALG-2 interacting protein X is a cytosolic calcium-binding protein that is required for cell death	Sperandio et al. (2004)
9	BKCa	The BKCa channel associated with mitochondria and ER was affected during the paraptosis	Ca2+ activated K+ channel (BKCa) channel	Hoa et al. (2007)
10	HMGB1	An important marker of paraptosis induction in T9 glioma cells, where they act as danger signals which can potentially stimulate an immune response	The translocation of HMGB1, a nuclear protein which executes various functions based on their location binding partners and their redox states on both sides of the cell	Hoa et al. (2009)
11	Prohibitin	Mediator of paraptosis	Inner mitochondrial protein involved in cell cycle regulation and tumour suppression	Sperandio et al. (2010)
12	cMYC/(TRIB3)/P62+	Activates paraptosis	c-MYC is a transcription factor that regulates cell growth, differentiation, metabolism and death. Increased c-MYC mediated the accumulation of tribbles homolog 3 (TRIB3)/P62+ aggresomes and consequently triggered paraptosis	Su et al. (2022)
13	TAJ/TROY	Activates	TAJ/TROY is a TNF receptor family member	Wang (2004)
14	IGF1R	Activates	Insulin-like growth factor I receptor (IGFIR)	Sperandio et al. (2000)
15	USP10	Regulator of paraptosis	Ubiquitin-specific peptidase 10, which specifically cleaves ubiquitin from ubiquitin-conjugated protein substrates	Kim et al. (2019a)
16	CBP/KU70/NOX2/BAX	Prevents paraptosis	Transcriptional network	Ding et al. (2021)
17	p20	Activates paraptosis	Activates Ca^2+^ mobilization in ER	Heath et al. (2012)
18	CLIC1	Activates paraptosis	Chloride intracellular channel-1 increases the intracellular Cl^−^ concentration	Zhu et al. (2019)

Recent reports on paraptosis induced in different cancer cells via ER stress have identified novel compounds and their mode of action. A nuclear receptor, Nur77, was found to bind with 4-(quinoline-4-amino) benzoyl hydrazide in hepatocellular carcinoma cells resulting in paraptosis via ER stress, vacuolation and autophagy (Li et al., 2022a). The oxy derivatives of disulfiram can inhibit the retrotranslocation of protein across the ER membrane to the cytosol, thus resulting in the accumulation of misfolded protein and ER swelling leading to paraptosis (Solovieva et al., 2022). The calcium release mediated by phospholipase C from ER through the IP_3_ receptor results in the onset of ER stress leading to cytoplasmic vacuolation, thus inducing the paraptotic cell death pathway (Pyrczak-Felczykowska et al., 2022). A non-antibiotic tetracycline, tetracycline-3 (4-dedimethylamino sancycline, COL-3), causes cell death in chronic myeloid leukemia cell line through DNA damage and initiates paraptosis characterized by mitochondrial and ER stress (Fares et al., 2022). High concentrations of DHW-221, a dual PI3K/mTOR inhibitor, induce a paraptotic effect in non-small lung cancer cells due to the activation of MAP kinase pathways and ER stress induction (Liu et al., 2022b). A polyphenolic flavonoid, Glabridin exhibits anti-tumour activity in breast cancer cell lines by inducing paraptosis in many human malignancies by elevating the expression of different ER stress markers like Bip, XBPIs, CHOP and by inducing ROS production and mitochondrial dysfunction (Cui and Cui, 2022).

### 4.3 ROS-mediated paraptosis

The dilation of mitochondria due to cristae disorganization takes place during paraptosis. Mitochondria, the storehouse of ATP production, is also the site for ROS production due to oxidative damage; therefore, a decrease in the mitochondrial membrane potential results in increased ROS generation. The anti-cancer activity of many compounds is via increased ROS production and ER stress pathway activation (Huang et al., 2017). Natural compounds like withaferin (Ghosh et al., 2016), morusin (Xue et al., 2018), curcuminoid B3 (Chen et al., 2019), jolkinolide B (Sang et al., 2021), etc., trigger ROS-induced paraptotic cell death in cancer cells. Recent studies have reported ROS induction can be increased in cancer cells by targeting thioredoxin reductase 1 (TrxR1) and by depleting the cellular glutathione (GSH) level (in Figure 4) (Chen et al., 2019; Zhao et al., 2023).

### 4.4 Ca^2+^ influx triggers mitochondria-mediated paraptosis

The calcium storehouse in the cell is ER which is composed of clusters of channels like IP_3_ receptors (IP3R) in the mitochondria-associated ER membranes (MAMs) (Patergnani et al., 2011). The cellular Ca^2+^ overload or redistribution of Ca^2+^ can lead to cell death (Zhivotovsky and Orrenius, 2011). Compounds such as celastrol (Yoon et al., 2014b), curcumin (Yoon et al., 2012), and hesperidin (Yumnam et al., 2014) have been shown to cause the release of calcium ions from the ER stores to the mitochondria, where its overload leads to the enlargement of the organelle. Calcium ions exit the ER via the IP3R and enter the mitochondria through the mitochondrial uniporter (MCU). Expression levels of both these receptors are increased during paraptosis, which facilitates the transport of calcium ions. The simultaneous dilation of both ER and mitochondria during paraptosis indicates the interconnection between the two organelles. Increased Ca^2+^ overload in the mitochondria causes loss in mitochondrial membrane potential followed by ROS generation and cell death (Lee et al., 2016; Yumnam et al., 2016; Kim et al., 2021; Raimondi et al., 2021). A mitochondrial Na^+^/Ca^2+^ exchanger inhibitor (CGP37157) has been identified to induce paraptosis in Jurkat cells through mitochondrial and ER membrane fusion that affects calcium metabolism and mitochondrial membrane potential (Yokoi et al., 2022).

The overload of Ca^2+^ results in the formation of swollen mitochondria, followed by fusion to form the mega mitochondria. ER undertakes the synthesis, folding and processing of proteins. Ca^2+^ is required for the functioning of the chaperones. Thus, their depletion will trigger the accumulation of misfolded proteins within the ER by impairing chaperone activity and protein processing (Michalak et al., 2002; Brostrom and Brostrom, 2003). This accumulation of misfolded proteins in the ER contributes to ER swelling. Mitochondrial Ca^2+^ overload has been shown to induce oxidative stress (Patergnani et al., 2011). Yoon et al. proposed that there may be feed-forward and self-amplified regulation between ROS generation and mitochondrial Ca^2+^ overload. They showed that the Ca^2+^ overload was upstream of the ROS generation and that ROS could further influence the IP3R to release Ca^2+^ resulting in a continuous cycle of reactions (Yoon et al., 2012). ROS also elevates ER stress, confirming the damage caused to ER and mitochondria. Mitochondrial Ca^2+^ overload causes an imbalance in the ion distribution in the organelle leading to a decrease in the membrane potential and collapse of the organelle. The increase in ROS can lead to stimulation of NADPH-P450 reductase (NPR) and hemoxygense-2 (HO), releasing carbon monoxide (CO), activating the BKCa channel followed by K^+^ and Na^+^ fluctuation within the cell. Due to the mitochondrial dysfunction induced by ROS and BKCa opening, the activity of ATP-dependent Na^+^ transporters exhausts the ATP pool and causes cell death, as shown in Figure 4 (Shubin et al., 2016). In summary, during paraptosis, the mitochondria and ER get impaired.

In paraptotic cells, the mitochondrial proteins such as ATP synthase and prohibitin increased. Inhibition of the F1F0 ATPase with oligomycin B prevented death, explaining the mitochondrial role in paraptosis. Prohibitin acting as a mediator is known for its role in cell-cycle regulation, replicative senescence, cellular immortalization, apoptosis and tumour suppression, highlighting its role as an agonist in the paraptotic death pathway (Sperandio et al., 2010).

### 4.5 Opening of ion channels induce paraptosis

Different ion channels play an essential role in the regulation of paraptosis. The pore-forming proteins which are present on membranes are called ion channels. They facilitate the transport of ions. Various types of ion channels have been identified, broadly categorized into voltage-gated and ligand-gated ion channels (Alexander et al., 2011).

The transport of calcium and the flow of calcium between ER and mitochondria play a vital role in regulating paraptosis. The dilation of mitochondria and ER during paraptosis can also be highly attributed to this calcium transport across membranes. Likewise, inositol 1,4,5, -triphosphate and ryanodine receptors have been reported to play a significant role in calcium transport during paraptosis induced by different compounds (Kim et al., 2021). The acidification due to the inhibition of the Na^+^/H^+^ exchanger resulted in a caspase-independent cell death that biochemically not morphologically resembles paraptosis (Schneider et al., 2004).

The studies conducted by Hoa et al. (2009) showed that opening potassium channels could lead to paraptosis by cell swelling. There can be pumping out of K^+^ ions when cells are subjected to ROS, resulting in the opening of the BK channels in the plasma membrane and ER and mitochondrial membrane. Due to this, Na^+^ and water get into cells so that the electric neutrality inside the cells is maintained, and this phenomenon further contributes to the swelling of cells. This swelling leads to cell lysis, releasing ‘danger signals’ like heat shock proteins and high mobility group B-1 (HMGB-1/amphoretin) protein, as in Figure 4.

A contradictory report suggested that the closure of big potassium calcium (BKCa) channels will lead to cytoplasmic swelling. They hypothesized that because of closed BKCa channels, the release of K^+^ ions would be prohibited, thus leading to the entry of water into the cells, further resulting in vacuole formation. As a result of the inhibition of the BKCa channel, the intracellular Ca^2+^ ions increased, resulting in membrane depolarization. Ophiobolin A, a fungal metabolite, inhibited the proliferation and migration of Glioblastoma multiforme cells by blocking the activity of the big conductance Ca^2+^-activated K^+^ channel (BKCa) channel. It was also observed that the BKCa channel associated with mitochondria and endoplasmic reticulum was affected during the paraptosis induced by the compound (Bury et al., 2013).

The role of chloride intracellular channel 1 (CLIC1) during paraptosis was identified when studying the ability of *Pharbitidis Semen* (RFP) to induce paraptosis. RFP-induced cell death was associated with the activation of the chloride channel and elevated intracellular chloride accumulation. The study also suggested that the blockage of the channel with DIDS (disodium 4,4'-diisothiocyanato-2,2'-stilbenedisulfonate hydrate) inhibited the cell death process (Zhu et al., 2019).

## 5 Modulators of paraptosis

Modulators are important for the regulation of function. Any alterations in this can lead to the activation or inactivation of different signalling pathways. Understanding different modulators involved in paraptosis will help us increase the chance of controlling cancer by initiating cell death. Proteomic analysis of cells undergoing paraptosis indicated a change in the expression and subcellular distribution of proteins involved in structural maintenance, signal transduction and cellular metabolism (Sperandio et al., 2010). A careful literature review lists more modulators or proteins that could directly or indirectly help in paraptosis. We have categorized the known proteins involved in paraptosis as activators, inhibitors, or regulators of this unique cell death pathway below and in Table 2.

### 5.1 Cytoskeletal proteins

The extensive cytoplasmic vacuolation and shrinkage of the cells observed during paraptosis were suggestive of the alterations in the cytoskeletal proteins. The head-to-tail lateral association of α/β heterodimers forms polar cytoskeletal filaments called microtubules, which must occur as single tubes and or cellular structures like a mitotic spindle and interphase networks (Hammond et al., 2008). Tropomyosin, the rod-like dimers synchronize the access of actin-binding protein to the filament by forming head-to-tail polymers along the length of actin filaments. It is clear from microscopic studies that paraptotic cells show alterations in structural arrangements, like rounding up the cells and the appearance of vacuoles in the cytoplasm. Cells undergoing paraptosis show elevated levels of disorganized microtubules due to changes in alpha and beta tubulin and tropomyosin, as shown by confocal microscopic studies followed by immunofluorescence. Cells at a more advanced stage of paraptosis showed a drastic decrease in both α- and β-tubulin levels. Thus, the uncanny redistribution of α- and β-tubulin and tropomyosin in the preliminary stages of paraptosis can thus be regarded as a stipulative marker of this cell death pathway (Sperandio et al., 2010). Studies have also reported that the F-actin (fibrillar actin) shrinks in amount without any alteration in the globular actin (Yang et al., 2015). These cytoskeletal reorganizations are the cause of the morphological changes seen during paraptosis.

### 5.2 High mobility group box-1 (HMGB1)

The osmotic lysis of paraptotic cells releases intracellular constituents like ATP, UTP, several proteases, heat shock proteins, high mobility group box-1 (HMGB1) etc. These molecules act as danger signals, playing a major role in stimulating cell-mediated immunity. HMGB1, a nuclear protein, executes various functions based on its location, binding partners, and their redox states (Hoa et al., 2009). HMGB1, when translocated to the periphery serves as an important marker of paraptosis induction in T9 glioma cells, where they act as danger signals which can potentially stimulate immune responses (Sperandio et al., 2010).

### 5.3 Prohibitin

Since mitochondria are involved in paraptosis, researchers have focused on identifying mitochondrial proteins over-expressed during paraptosis; one protein identified is prohibitin (Sperandio et al., 2010). Prohibitin is a protein that plays an important role in regulating p53 activity, serving as a switch between proliferation and apoptosis (Fusaro et al., 2002).

### 5.4 Ubiquitin specific peptidase 10 (USP10)

To identify the genes responsible for curcumin-induced paraptosis in malignant breast cancer cells, siRNAs that can block curcumin-induced mitochondrial dilation were screened from the siRNA library using CHOP siRNA as a positive control. USP10, which is a cytoplasmic ubiquitin-specific protease, has been found to have a role in cell death.

During DNA damage, they deubiquitinate p53, which helps reverse the nuclear export and degradation by Mdm2. The role of USP10 in inducing mitochondrial alterations during curcumin-induced paraptosis was identified by the knockdown of USP10 by the specific siRNA and with their specific inhibitor (spastin-1) (Kim et al., 2019a).

### 5.5 Caspase-9

Even though being a caspase-independent form of cell death, studies by Sperandio et al. (2000) have reported that caspase 9 can be an effective activator of paraptosis. In Lower-Grade Gliomas (LGG) prognostic model, ten paraptosis-related gene (PRG) signatures (CDK4, TNK2, DSTYK, CDKN3, CCR4, CASP9, HSPA5, RGR, LPAR1, and PDCD6IP) were identified to categorize LGG patients into high- and low-risk subgroups (Qian, 2022).

### 5.6 Ku70-NOX2-CBP

Ding et al. (2021) studied the role of an acetyltransferase, CBP (CREB binding protein), in human melanoma in preventing paraptosis, necrosis, and apoptosis. The importance of ROS in redox homeostasis has been widely studied, and its loss of balance can alter the cytotoxic effects of ROS, which can cause chromosomal degradation, genetic instability and act as an etiological factor in many diseases such as neurodegeneration, cardiovascular defects, oncogenesis, metabolic dysregulation, chronic inflammation, organ damage, etc. They found that the depletion of either CBP or Ku70 increased the expression of the NOX2 gene, affecting the transcription of phase II detoxification enzymes via the Nrf2-Keap pathway resulting in increased ROS generation. This ROS increase induced cytoplasmic vacuolation, S phase cell cycle arrest, necrosis and paraptotic cell death. Knockdown of CBP also increased Bax expression in the cytoplasm, causing apoptotic cell death. Therefore, this transcriptional network plays an essential link in the ROS-mediated oxidative environment that helps in melanoma proliferation by preventing cell death pathways like paraptosis, necrosis, and apoptosis (Ding et al., 2021).

### 5.7 p20

Bap31 is an ER-localized polytopic transmembrane protein that helps in escorting proteins. The cleavage of Bap31 by caspase-8 results in the generation of a membrane-embedded proapoptotic fragment known as p20Bap31. p20 expression was shown to be generally initiated by the activation of Ca^2+^ mobilization in ER. Hannah et al. (2012) studied the role of Bax/Bak in p20-induced cell death pathway in wild-type and Bax/Bak double knockout (DKO) baby mouse kidney immortalized epithelial cell lines. Interestingly, they found that p20 initiated a paraptosis-like cell death pathway characterized by ER dilation independent of Bax/Bak, which can be delayed by the increased expression of ER-restricted Bcl2 in DKO cells (Heath-Engel et al., 2012).

### 5.8 Phosphatidylethanolamine binding proteins (PEBP)

Phosphatidylethanolamine binding protein (PEBP), a highly conserved multifunctional protein present in bacteria to human (He et al., 2016). Studies conducted by Sperandio et al. (2010) first reported the inhibitory effect of PEBP-1 on paraptosis by conducting 2D gel electrophoresis and Mass spectrometric analysis.

### 5.9 AIP1/Alix

Another identified inhibitor is the Alix (ALG-2 interacting protein X), a cytosolic calcium-binding protein required for cell death (Trioulier et al., 2004). The inhibitory role of Alix was put forward by Sperandio *et al.* (Sperandio et al., 2004). A prenylated flavonoid: Epimedokoreanin B (EKB) can induce paraptosis in non-small lung cancer (A549) and NCI-H292 by the induction of cytoplasmic vacuolation. EKB-induced death was associated with the downregulation of Alix (Zheng et al., 2022).

### 5.10 Autophagic markers

Autophagy is a highly conserved process that targets aggregated proteins, pathogens, damaged organelles, and macromolecules for lysosome degradation. The formation of double membrane structures is the initiation step of autophagy. A well-known autophagy marker is the LC3-II (Microtubule-associated protein light chain 3). They are generated by the conjugation of cytosolic LC3-I to phosphatidylethanolamine on the surface of autophagosomes. Another important marker of autophagy is the Sequestosome 1 (SQSTM 1)/p62 which is a multidomain protein that can bind both to ubiquitinated proteins and LC3-II through the Ub-associated domain and LC3-interacting region respectively (Cohen-Kaplan et al., 2016).

Many studies pointed out that during paraptosis, the ubiquitinated proteins are engulfed in the autophagosome, but autophagosomal-lysosomal fusion does not happen, therefore there is no decrease in the level of autophagic marker proteins like LC3B and p62 (Zheng et al., 2022). Autophagy is characterized by the fusion of autophagosome, and lysosome. An initial increase in LC3B and p62 is followed by a decrease due to lysosomal fusion-mediated degradation (Pankiv et al., 2007). Therefore, an increase in the level of autophagic markers p62 and LC3BII is used to detect paraptosis. Adequate reports are suggesting the high-level occurrence of both the autophagic marker proteins during paraptosis. During 6-Shogaol-induced paraptosis, there is an elevated expression of both LC3B and p62, shown by Western blotting analysis (Nedungadi et al., 2018). The upregulation of microtubule-associated light chain protein 3B was reported during paraptosis in MDA-MB-231 and MCF-7 cell lines on treatment with pyrazolo[3,4-h]quinoline scaffold derivative, accompanied by the downregulation of Alix, elevated ROS production, c-Jun N terminal Kinase, and ER stress (Nguyen et al., 2022). Moreover, it is unlikely that autophagy markers would be appropriate markers of different patterns of paraptosis in Jurkat cells (Balachandran et al., 2021).

### 5.11 HSP70

HSP70 is a molecular chaperone that is involved in proper folding and sorting (Mayer and Bukau, 2005). Inhibition of HSP70 using VER155008 (HSP70 inhibitor) induced paraptosis in thyroid carcinoma cell line (Kim et al., 2014). Combinatorial treatment of geldanamycin and Velcade results in the simultaneous inhibition of Hsp 90 and proteasome followed by the accumulation of ER-mediated vacuolation along with the disruption of (valosin-containing protein) VCP mediated regulation of ER secretory pathway (Mimnaugh et al., 2006).

### 5.12 Chloride intracellular channel-1 (CLIC1)

An increase in the intracellular Cl^−^ concentration by the activation of chloride intracellular channel-1 (CLIC1) induced paraptosis by a purified resin glycoside fraction (RFP) of *Pharbitidis Semen* in human colon cancer cell line, thus suggesting CLIC1 as another channel playing a role in paraptosis (Zhu et al., 2019).

## 6 Recent trends in paraptosis research

### 6.1 Natural compounds inducing paraptosis

**FIGURE 5:**
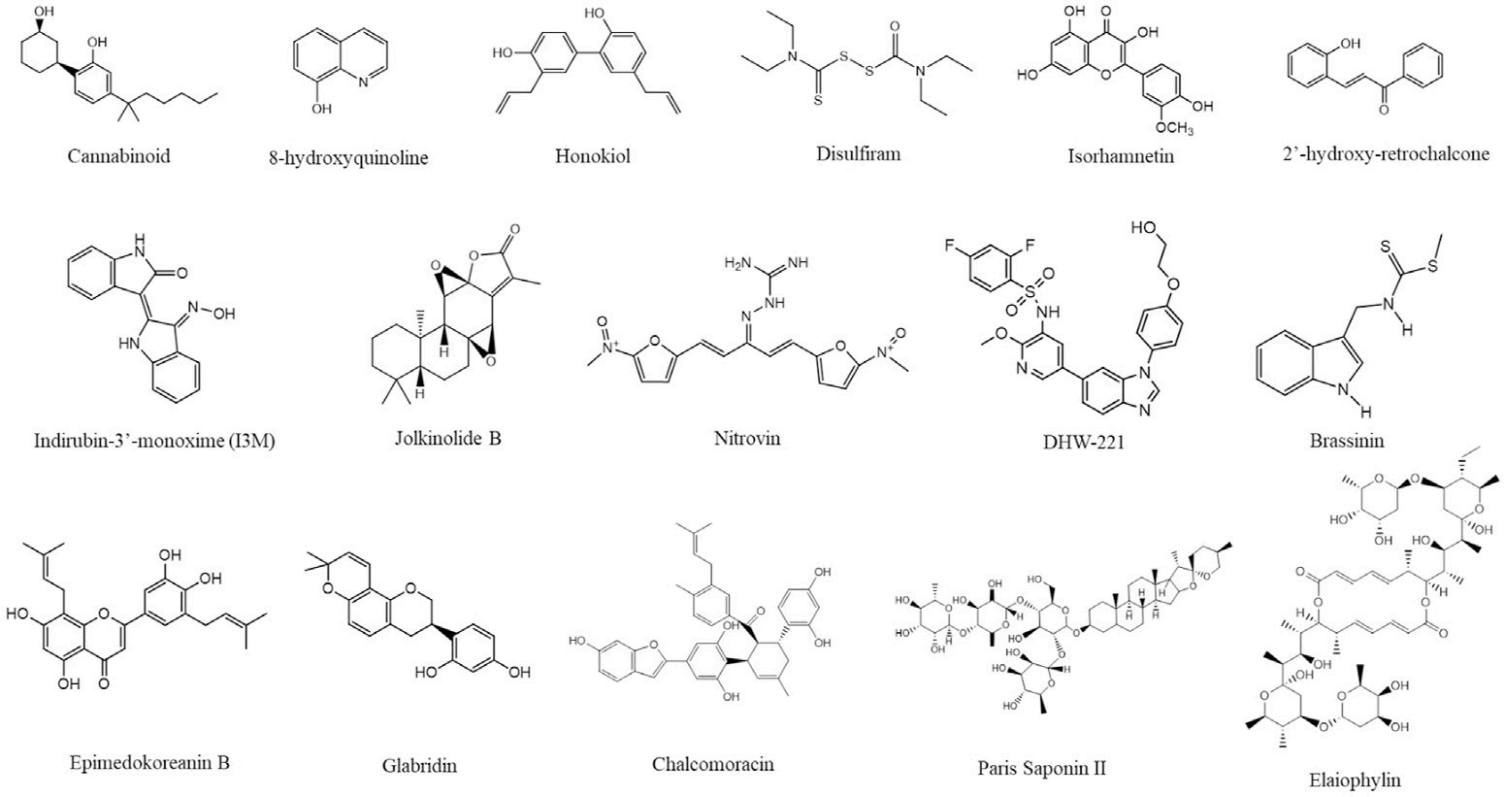
Structure of different paraptosis inducing natural compounds. The structures were drawn using ChemDraw (https://perkinelmerinformatics.com/products/research/chemdraw) and the figure was generated using Canva (www.canva.com).

Different natural products and small molecules that induce paraptosis have been reviewed in several articles (Lee et al., 2016; Wang et al., 2018; Fontana et al., 2020). Recently reported natural compounds from 2020 to 2023 are shown in Figure 5. Among them, the structure of isoxazole derivative of usnic acid (Pyrczak-Felczykowska et al., 2022) was not available.

**FIGURE 6:**
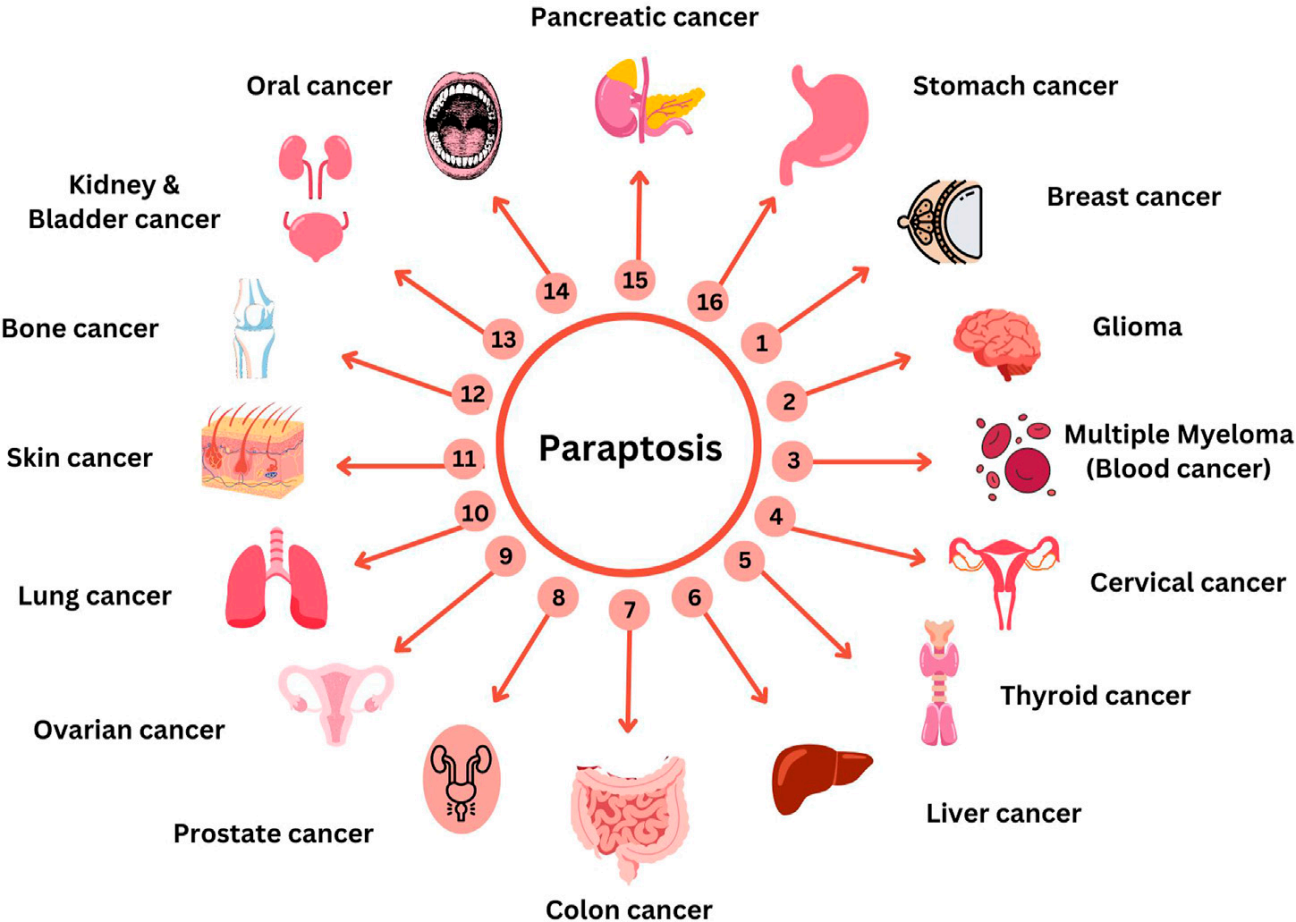
Compounds targeting paraptosis in different cancer. This figure was generated using Canva (www.canva.com).

Secondary metabolites from natural sources are known for their role in inducing paraptotic cell death in various cancer cells by targeting different pathways (Wang et al., 2018). Ginger is known for its anti-cancer properties due to the presence of phenolic compounds like gingerol, shogaol, paradol, etc. Both ginger ethanolic extract and purified 6-shogaol were found to induce paraptosis in breast cancer via two different mechanisms (Nedungadi et al., 2018; Nedungadi et al., 2021). This brings in the possible importance of paraptosis in alternative plant-based therapies for cancer through Ayurveda, Unani and Homeopathy where a mixture of natural products are used either directly or after processing. In this review, we have collected all the natural compounds inducing paraptosis and arranged them according to different cancer types, cell lines, and mechanisms, as mentioned in Table 3. The different cancer cell types susceptible to paraptotic cell death are depicted in Figure 6. There are also studies being reported that various natural compounds can induce a synergistic effect on cancer cells by inducing both paraptosis and apoptosis (Korsnes, 2012; Kayacan et al., 2021; Li et al., 2021; Ma et al., 2022).

**TABLE 3:** Paraptosis-inducing compounds against cancer cell lines.

Sl. No:					Compounds	Cell line type								Cell line						Signalling pathway and mechanism						References		
1. Breast																												
i)					Curcumin	Melanocyte								MDA-MB-434S						Inhibition of mitochondrial Na^+^/Ca^2+^ exchanger (mNCX) and proteasome, pERK1/2↑, p-JNKs↑, Alix↓						Yoon et al., 2010 (2012)		
						Epithelial								MDA-MB-231, HS578T														
ii)					Dimethoxy curcumin	Melanocyte								MDA-MB-434S						Proteasomal inhibition and ER stress, pERK1/2↑, p-JNKs ↑, Alix↓						Yoon, Kang, et al. (2014a)		
						Epithelial								MDA-MB-231, HS578T, MCF-7														
iii)					Celastrol	Melanocyte								MDA-MB-434S						Ca^2+^ overload, proteasomal inhibition via ER stress, pERK1/2↑, p-JNKs ↑, p-p38						Yoon et al. (2014)		
						Epithelial								MCF-7														
iv)					15d-PGJ2	Epithelial								MDA-MB-231						Disruption of sulfhydryl homeostasis, ER stress, pERK1/2↑						Kar et al. (2009)		
v)					Manumycin A	Epithelial								MDA-MB-231, BT-20						ER stress, accumulation of ubiquitinated proteins, p21↑, p27 ↑, PTEN ↑						Singha et al. (2013)		
						Lymphoblast								HCC1937														
vi)					Withaferin A	Epithelial								MDA-MB-231, MCF-7						ER stress, ROS production, Alix↓						Ghosh et al. (2016)		
vii)					Deoxyelephantopin derivative (DETD)	Epithelial								MDA-MB-231						Oxidative and ER stress, p-JNK↑						Shiau et al. (2017)		
viii)					Chalcomoracin	Epithelial								MDA-MB-231						ROS production, PINK1 ↑, Alix ↓, p-ERK↑						Han et al. (2018)		
ix)					6-Shogaol	Epithelial								MDA-MB-231						Proteasomal inhibition, ER stress						Nedungadi, et al. (2018)		
x)					Plumbagin	Epithelial								MDA-MB-231						Disruption of sulfhydryl homeostasis and inhibition of proteasome						Binoy and Nedungadi (2019)		
xi)					2′-hydroxy-retrochalcone	Epithelial								MDA-MB-231						Proteasomal dysfunction and ER stress						Nedungadi et al. (2021)		
xii)					Indirubin-3′-monoxime (I3M)	Epithelial								MDA-MB-231						Proteasomal dysfunction and ER stress-mediated Ca^2+^ release.						Dilshara et al. (2021)		
xiii)					Cannabinoids (C6 combination)	Epithelial								MDA-MB-231, MCF-7						ER stress (GRP78 increase)						Schoeman et al. (2020)		
xiv)					Gambogic Acid	Epithelial								MDA-MB-453, MDA-MB-468, MDA-MB-435S						Disruption of thiol proteostasis						Seo et al. (2019)		
						Melanocyte																						
xv)					5,7-dibromo-8-(methoxymethoxy)-2-methylquinoline (HQ-11)	Epithelial								MDA-MB-231, MCF-7						ER stress, proteasomal inhibition, pERK↑						Ma et al. (2022)		
xvi)					Glabridin	Epithelial								MDA-MB-231, MCF-7						ER stress, poly ubiquitinated protein accumulation, proteasome suppression, ROS production, MMP loss						Cui & Cui (2022)		
xvii)					Isoxazole derivative of usnic acid	Epithelial								MDA-MB-231, MCF-7						ER stress, IP3R channel activation						Pyrczak-Felczykowska et al. (2022)		
xviii)					Derivative of pyrazolo[3,4-*h*]quinoline scaffold (YRL1091)	Epithelial								MDA-MB-231, MCF-7						ER stress, accumulation of ubiquitinated proteins, ROS production, ERK↑, JNK↑, Alix↓						Nguyen et al. (2022)		
xix)					Ginger extract	Epithelial								MDA-MB-231						ER stress, mitochondrial dysfunction, AIF translocation and DNA damage						Nedungadi et al. (2021)		
xx)					Disulfiram oxy-derivatives	Epithelial								MCF-7						ER stress, mitochondrial damage, 20S proteasome inhibition and actin depolymerization at later stages						Solovieva et al. (2022)		
2. Brain																												
i)					Curcumin	Glioblastoma								A172						via microRNAs, AKT-Insulin, and p53-BCL2 networks, and AKT protein level reduction was confirmed						Garrido-Armas et al. (2018)		
ii)					Ophiobolin A	Pleomorphicastrocytoid, Neuronal, Fibroblast, Fibroblast) Fibroblast								U373-MG, U251N, U251MG, A172						ER stress, NAC inhibition, decrease of BKCa channel						Bury et al. (2013)		
														T98G														
iii)					Oligomeric Procyanidins	Epithelial								U-87						Extracellular Ca^2+^ influx, pERK1/2↑, p-p38 ↑						Zhang et al. (2010)		
iv)					Paclitaxel	Epithelial								U-87						CHX has no effect, MEK, p38 and JNK pathways are not involved						Sun et al. (2010)		
v)					Yessotoxin	Muscle cells from intracranial tumor								BC3H1						ER and mitochondrial swelling, p-JNK↑						Korsnes et al. (2011)		
vi)					1-Desulfo Yessotoxin	Muscle cells from intracranial tumor								BC3H1						ER and mitochondrial swelling, p-p38↑						Korsnes et al. (2013)		
vii)					Xanthohumol	Epithelial								SH-SY5Y						ER stress and LC3B upregulation, p38 ↑						Mi et al. (2017)		
3. Blood																												
i)					Honokiol	Lymphoblast								K562						ROS generation ROS generation, ER stress, LC3 upregulation, mTOR and MAPK activated						Liu et al. (2021), Wang et al. (2013)		
						Promyelocyte								NB4														
ii)					Xanthohumol	Promyeloblast								HL-60						ER stress and LC3B upregulation, p38 ↑						Mi et al. (2017)		
iii)					Iturin lipopeptide	Lymphoblast								K562						LC3B and p62 upregulation						Zhao et al. (2019)		
iv)					Brassinin	Lymphoblast								K562						ROS production, mitochondrial damage, ER stress, and activation of MAPK						Yang et al. (2023)		
						Lymphoblast-like								KBM5, LAMA84, and KCL22														
4. Cervical																												
i)					Celastrol	Epithelial								HeLa						Proteasome inhibition, Mitochondrial Ca^2+^ overload, pERK1/2↑, p-JNKs ↑, p-p38 ↑						Wang et al. (2012)		
ii)					Cyclosporin A	Epithelial								HeLa, SiHa						LC3 upregulation, Cyclophilin B↓, Alix↓						Ram and Ramakrishna (2014)		
iii)					8-p-Hydroxybenzoyl tovarol	Epithelial								HeLa						Bip, CHOP, IRE1α and XBP1 upregulation						Zhang et al. (2015)		
iv)					Seleno-DL-Cystine	Epithelial								HeLa						Bip and CHOP polyubiquitination upregulation, ROS generation						Wallenberg et al. (2014)		
v)					Paclitaxel	Epithelial								HeLa						CHX has no effect, MEK, p38 and JNK are not involved						Sun et al. (2010)		
vi)					Wheat germ Agglutinin	Epithelial								HeLa, SiHa, CaSKi						Autophagy-linked FYVE (Alfy) protein inhibition, ER stress, LC3B upregulation						Tsai et al. (2017)		
vii)					2′-hydroxy-retrochalcone	Epithelial								HeLa						Proteasomal dysfunction, ER stress, LC3 upregulation						Nedungadi et al. (2021)		
5. Thyroid																												
i)					Tunicamycin	Epithelial								8505C, CAL62, FRO cell lines						Bip, CHOP, p-PERK and IRE1 upregulation						Kim et al. (2014)		
6. Liver																												
i)					Hesperidin	Epithelial								HepG2						Mitochondrial dysfunction and Ca^2+^ overload, p-ERK↑						Yumnam et al. (2016)		
ii)					Cis-Nerolidol	Epithelial								HepG2/C3 A						ER stress, increased activity of cytochrome P450 enzymes						Biazi et al. (2017)		
iii)					Gambogic Acid	Epithelial; diffusely spreading cells								SNU-449						Proteasomal inhibition and ER stress, ROS independent- mitochondrial depolarization						Seo et al. (2019)		
7. Colon																												
i)					Curcumin	Epithelial								HCT116						Proteasome inhibition ROS, Mitochondrial Ca^2+^ overload, LC3 upregulation, pERK1/2↑, p-JNKs↑, Alix↓						Lee et al. (2015)		
ii)					Celastrol	Epithelial								DLD-1, RKO						Proteasome inhibition, Mitochondrial Ca^2+^ overload, pERK1/2↑, p-JNKs ↑, p-p38 ↑						Yoon et al. (2014)		
iii)					15d-PGJ2	Epithelial								HCT116						Disruption of sulfhydryl homeostasis LC3 upregulation, pERK1/2↑						Kar et al. (2009)		
iv)					Ginsenoside Rh2	Epithelial								HCT116, SW480						p53 activation, activation of death by antioxidants						Li et al. (2011), Wan et al. (2018)		
v)					Protopanaxadiol	Epithelial								HCT116, SW480						Death acceleration by inhibiting ROS generation, NF-κB activated						Wang et al. (2013)		
vi)					ɣ-Tocotrienol	Epithelial								SW620 and HCT-8						Wnt signals↓ (β-catenin, cyclin D, c-Jun)						Zhang et al., (2011) (2013)		
					δ-Tocotrienol	Epithelial								SW620						Wnt signals↓ (β-catenin, cyclin D, c-Jun)								
vii)					Iturin A-like lipopeptides	Epithelial								Caco-2						ER stress, ROS generation, Ca^2+^ ↑						Zhao et al. (2019)		
viii)					Loperamide	Epithelial								DLD-1, SW-480, SW-620, HCT116						ER stress, Ca^2+^ imbalance and CHOP↑						Kim et al. (2019)		
ix)					Purified resin glycoside fraction (Pharbitidis Semen)	Epithelial								HT-29 and HCT-116						Chloride intracellular channel-1 activation and intracellular Cl^−^↑, MAPK activation						Zhu et al. (2019)		
8. Prostate																												
i)					Curcumin	Epithelial								PC-3M						Proteasome inhibition ROS, Mitochondrial Ca^2+^ overload, LC3 upregulation, pERK1/2↑, p-JNKs↑, Alix↓						Lee et al. (2015)		
ii)					15d-PGJ2	Epithelial								DU145						Disruption of sulfhydryl homeostasis LC3 upregulation, pERK1/2↑						Kar et al. (2009)		
iii)					Benzo[a]quinolizidine analogs	Epithelial								PC3						ER stress and LC3B upregulation						Zheng et al. (2016)		
iv)					Chalcomoracin	Epithelial								LNCaP, PC-3						ROS generation, ER stress, PINK1 ↑, Alix ↓, p-ERK↑						Han et al. (2018)		
v)					δ-Tocotrienol	Epithelial								CRPC cells—DU145, PC-3						ER stress, LC3 and p62 upregulation, p-JNK ↑, p-p38 ↑						Fontana et al. (2020)		
9. Ovarian																												
i)					Morusin	Epithelial								A2780, HO-8910, SKOV3						Ca^2+^ overload, ROS generation and loss of mitochondrial membrane potential						Xue et al. (2018)		
ii)					Elaiophylin	Epithelial								SKOV3, OVCAR8, UWB1.289, SW626						ER stress, SHP2/SOS1/MAPK↑						Li et al. (2022)		
10. Lung																												
(i)					Cyclosporin A	Epithelial								A549						LC3 upregulation, Cyclophilin B↓, Alix↓						Ram and Ramakrishna (2014)		
ii)					Paclitaxel	Epithelial								A549						CHX has no effect, MEK, p38 and JNK are not involved						Guo et al. (2010)		
						Epithelial								ASTC-a-1														
iii)					6-Shogaol	Epithelial								A549						Proteasome inhibition, ER stress, ROS generation, LC3 upregulation						Nedungadi et al. (2018)		
iv)					Hinokitiol copper complex	Epithelial								A549						Proteasome inhibition, ER stress						Chen et al. (2017)		
v)					Chalcomoracin	Epithelial								H460						ER stress, MAPK activation						Han et al. (2018)		
						Epithelial								A549														
						Adenocyte								PC-9														
vi)					Paris Saponin II (PSII)	Epithelial								NCI-H460						ER stress, JNK pathway activation						Man et al. (2020)		
						Epithelial								NCI-H520														
vii)					Prenylated bibenzyls (*Radula constricta*)	Epithelial								A549, NCI-H1299						ROS elevation and loss in mitochondrial membrane potential						Zhang et al. (2019)		
viii)					Gambogic Acid	Epithelial								NCI-H460						Proteasomal inhibition and ER stress, ROS independent- mitochondrial depolarization						Seo et al. (2019)		
ix)					Epimedokoreanin B	Epithelial								A549, NCL-H292						ER stress, autophagosome accumulation, ROS production, loss of MMP, UPR signaling						Zheng et al. (2022)		
x)					DHW-221	Epithelial								A549						ER stress, PI3K/mTOR inhibition, MAPK activation						Liu et al. (2022)		
xi)					Ginger extract	Epithelial								A549						ER stress, mitochondrial dysfunction, AIF translocation and DNA damage						Nedungadi et al. (2021)		
11. Skin																												
i)					Cyclosporin A	Keratinocyte								HaCaT						LC3 upregulation, Cyclophilin B↓, Alix↓						Ram and Ramakrishna (2014)		
ii)					δ-tocotrienol	Epithelial								A375						Ca^2+^ overload and ROS generation, MAPK activation						Raimondi et al. (2021)		
12. Bone																												
i)					Cyclosporin A	Epithelial								U2OS, Saos-2						LC3 upregulation, Cyclophilin B↓, Alix↓						Ram and Ramakrishna (2014)		
13. Kidney and Bladder																												
i)					Jolkinolide B	Epithelial								T24, UM-UC-3, T24/CDDP						ROS-mediated ER stress, MAPK and ERK activation						Sang et al. (2021)		
14. Oral																												
i)					Isorhamnetin (3′-Methoxy-3,4′,5,7-tetrahydroxyflavone)	Epithelial								HSC-3, HSC-4, PE/CA-PJ15						↑ROS generation, ERK/MAPK						Chen et al. (2021)		
15. Pancreas																												
i)					Gambogic Acid	Epithelial								BxPC-3						Proteasomal inhibition and ER stress, ROS- independent mitochondrial depolarization						Seo et al. (2019)		
16. Stomach																												
i)					Gambogic Acid	Epithelial								SNU-668 (gastric cancer)						Proteasomal inhibition and ER stress, ROS independent- mitochondrial depolarization						Seo et al. (2019)		

### 6.2 Mouse xenograft model

Animal xenografts for human cancers have been implied as a tool for pre-clinical studies where mouse xenografts are the gold standard models that are widely used for *in vivo* studies due to their features like the presence of comparable genome size with humans, short reproductive phase, large litter size, cost-effective and easy maintenance (Lee et al., 2018). Studies have been reported that show the effect of different compounds along with a few combinatorial approaches inducing paraptosis-mediated cell death in xenograft mouse models. Many combinatorial treatments like celastrol-afatinib (Dai et al., 2021), paclitaxel-honokiol (Li et al., 2021), Jolkinolide B and mTORi (Sang et al., 2021) have been reported to induce paraptotic cell death in cancer. The xenograft tumour model introduces a therapeutic strategy for drug resistance. ^125^I radiation has been shown to induce different cell death mechanisms like apoptosis, autophagy and paraptosis via ROS generation in xenograft model (Wang et al., 2020). The effect of phosphino copper (I) complex in triggering UPR pathway-mediated paraptosis in both drug-resistant and sensitive murine models (Gandin et al., 2017); and other studies defining the efficacy of different compounds inducing paraptotic cell death in various cancer have also been reported (Wang and Chen, 2012; Zhao et al., 2021) (Figure 6). The issue of multiple drug resistance in multiple tumour-bearing mice models against platinum, taxane, and PARPi could be overcome by inducing the hyperactivation of an upstream modulator of MAP kinase pathway, SHP2 by a bacterial compound named Elaiophylin (Li et al., 2022a). Studies were also done in zebrafish xenograft model where the role of Epimedokoreanin B (EKB) in inducing paraptosis through the downregulation of Alix, and upregulation of ER stress markers proteins was identified (Zheng et al., 2022). In an orthotopic A549/Taxol tumour mouse model higher concentration of DHW-221, a dual PI3K/mTOR inhibitor could reinforce the paraptotic pathway through FOXO3a nuclear translocation (Liu et al., 2022c).

### 6.3 Photodynamic therapy (PDT)

PDT involves the use of photosensitizing agents accompanied by irradiation at a specific wavelength that matches the absorbance band of the sensitizer. A series of events occur as a result of exposure to oxygen which includes tumour cell death, microvasculature damage and local inflammatory reactions (Agostinis et al., 2011; Pierroz et al., 2016). PDT being a light-catalyzed process results in the generation of ROS at sub-cellular sites thereby initiating different cell death pathways. The cytoprotective role of autophagy occurs in these malignant cells exposed to photodynamic damage. As a result, targeting lysosome is found to be more effective in photokilling since autophagy cannot offer cytoprotection. The overall level of photokilling can be enhanced by targeting lysosome and mitochondria together for photodynamic damage (Kessel and Reiners, 2020). PDT targeting ER/mitochondria is found to initially induce paraptosis followed by apoptosis (Kessel, 2006; Mishchenko et al., 2022). Cancer cells can pass through various non-conventional cell death pathways, including paraptosis, parthanatos, mitotic catastrophe, pyroptosis, necroptosis, and ferroptosis when exposed to PDT. PDT being an anti-cancer treatment method can trigger immunogenic cell death (ICD) (Mishchenko et al., 2022). PDT using PyroMor, a combination of Pyropheophorbide-a (Pyro) and morusin (Mor) is capable of inducing paraptosis through ER and mitochondrial vacuolization (Hoa et al., 2007).

### 6.4 Tumour vaccine through expression of M-CSF

Human monocytes/macrophages are found to kill human glioma cells (U251) expressing membrane macrophage colony-stimulating factor (mM-CSF) via paraptosis thus, proving its potential to be used as a safe live tumour cell vaccine (Hoa et al., 2007). mM-CSF expressing T9 glioma cells exhibited strong destruction of tumour by polymorphonuclear leukocytes via the induction of paraptosis (Chen et al., 2002). Three different forms of the heat shock proteins (HSPs), HSP60, HSP70 and GRP94 (gp96), associated with the induction of tumour immunity were found to be expressed in these mM-CSF-transfected tumour cells (Jadus et al., 2003). Along with these HSPs, translocation of HMGB1 from the nuclear region to the periphery occurs and these molecules are found to be “danger signals” that can stimulate immune responses (Hoa et al., 2009).

### 6.5 Nanomedicine

Anticancer therapy can be improved using nanomedicines. Liposomal doxorubicin (1995) was the first FDA-approved anticancer nanomedicine used based on its enhanced permeability and retention ability (Rasool et al., 2022). Potent paraptosis-inducing nanomedicines which can overcome cancer drug resistance have been reported. An amphiphilic 8-hydroxyquinoline (HQ) conjugate block copolymer with polyethylene glycol in the form of a micelle is reported to be used as a nanomedicine. Here, the HQ conjugation linker gets hydrolyzed by Cu, forming a complex Cu(HQ)_2_, a strong proteasomal inhibitor which induces paraptosis (Zhou et al., 2018). Pancreatic ductal adenocarcinoma was reported to be inhibited by the treatment with silver nanoparticles (AgNP) through the induction of paraptosis, by blocking the cell migration, spheroid and colony formation, cytoplasmic vacuolation, ROS generation, mitochondrial and ER dilation, MAP Kinase activity, LC3B, p62 expression (Liu et al., 2022a). A nanosized Cu^2+^-coordinated morusin/doxorubicin biological organizer (COMBO) has been designed for tumour therapy which could induce paraptosis along with apoptotic form of cell death (Zheng et al., 2021). Cu(DDC)_2_ nanoparticles (NPs), a combination of copper ions and DSF developed through a facile stabilized metal ion ligand complex (SMILE) method is reported to induce cell death in drug-resistant prostate cancer cells (DU145-TXR) through paraptosis (Chen et al*.*, 2018).

### 6.6 Metal complexes

Metal-based compounds have been used in the treatment of various types of cancer. Barnett Rosenberg in 1960 discovered cisplatin as a metal-based anti-cancer drug. The metal-based therapy already in use includes platinum drugs, such as cisplatin, carboplatin and oxaliplatin (Ndagi et al., 2017). Metal complexes have also been found to induce paraptosis in different cancer cells. Copper complexes like A0 (thioxotriazole copper(II) complex) reported to induce paraptosis through mechanisms involving eIF2alpha phosphorylation and the UPR (Tardito et al., 2009). More recently Hinokitiol copper complex (HK-Cu), is found to inhibit 19S proteasomal deubiquitinase (DUB) activity resulting in the accumulation of ubiquitinated proteins in A549 and K562 cells, thereby inducing paraptotic cell death. Thus, representing HK-Cu as a potential drug candidate for cancer treatment (Chen et al., 2017). Ye et al. (2015) identified phosphorescent rhenium (I) tricarbonyl polypyridine complex ReLMito ([Re(DIP)(CO)_3_(L)](PF_6_)) which specifically localizes mitochondria, inhibits the activity of HDACs. This molecule was further identified to induce paraptosis through mitochondrial membrane permeabilization and reactive oxygen species (ROS) generation. A series of platinum complexes with varying ligands (diimine and acyl thiourea) were synthesized and checked for anticancer activity. Changes in morphology indicate that these active platinum complexes induce cell death through apoptosis and paraptosis (Peega et al., 2021). Thiosemicarbazone Me_2_NNMe_2_, a copper complex is reported to induce paraptosis by inhibiting ER-resident protein disulfide isomerase, resulting in the disruption of the Ca^2+^ and ER thiol redox homeostasis-mediated ER stress (Hager et al., 2018). The antitumour activity of iridium complexes through cyclometalated iridium (iii) complexes which induce paraptotic cell death through a series of mitochondria-related dysfunctional events was reported (He et al., 2018). Induction of paraptosis by cyclometalated iridium (Ir) complex-peptide hybrid (IPH) and CGP37157 (an inhibitor of a mitochondrial Na^+^/Ca^2+^ exchanger) is also reported to be associated with membrane fusion between mitochondria and the ER, followed by Ca^2+^ influx from the ER to mitochondria, and a decrease in the mitochondrial membrane potential (*ΔΨ*m) (Yokoi et al., 2022). It has been reported that paraptosis induced by iridium complex-peptide hybrids and triptycene-peptide hybrids are inhibited by carbonyl cyanide 3-chloroophenylhydrazone (CCCP), while paraptosis induced by a natural product such as celastrol is not inhibited by CCCP (Yamaguchi et al., 2022; Yokoi et al., 2022).

### 6.7 Combinatorial therapy

Bortezomib (Bz) which induces integrated stress response (ISR) when combined with Integrated stress response inhibitor (ISRIB), drives the Bz insensitive breast cancer cell line towards paraptosis (Nguyen et al., 2022). mTOR inhibition by everolimus could induce caspase-independent cell death in acute lymphoblastic leukaemia cells (Baraz et al., 2014). A combination of mTOR inhibitor everolimus and ginsenoside Rh2 could trigger cytoplasmic vacuolation-mediated paraptosis (Su et al., 2022). The pro-apoptotic and pro-paraptotic activity of an mTOR inhibitor is elevated upon its combination with Jolkinolide B (JB) through the suppression of AKT feedback activation and cytoprotective autophagy (Sang et al., 2022). Paraptosis was induced in MCF-7 cells upon treatment with both diethyldithiocarbamate and B12b and if there is short exposure of DSFoxy along with this, it led to lysosomal cell death (Solovieva et al., 2022). A triptycene-peptide hybrid: syn-6 and anti-6 is capable of inducing paraptosis in Jurkat cells by the loss of mitochondrial membrane potential and cytoplasmic vacuolation (Yamaguchi et al., 2022). The membrane interaction between the poorly differentiated gastric adenocarcinoma, and tumour-associated tissue eosinophilia can result in the activation and degranulation of Eosinophil sombrero vesicles towards the tumour cells, which induced paraptosis in the tumour cells due to the formation of cytoplasmic vacuoles, and mitochondrial swelling (Caruso et al., 2022).

### 6.8 Activation of T-cell immune response through paraptosis

The effect of ablation of endoplasmic reticulum stress-related kinase, PERK, in melanoma cells has been investigated. They found that the deletion of PERK in melanoma cells activated sec61β-linked paraptosis by inhibiting UPR. Thus, the induction of paraptosis in the melanoma cells resulted in the release of damage-associated molecular patterns (DAMPs) like HMGB1 and ATP. T-cell immunity gets activated by promoting monocytic-lineage inflammatory dendritic cells via type-I interferon, IFN-STAT1. Thus paraptosis induction in cancer cells can increase the T-cell response-mediated anti-tumour immunity (Mandula et al., 2022).

## 7 Discussion

**FIGURE 7:**
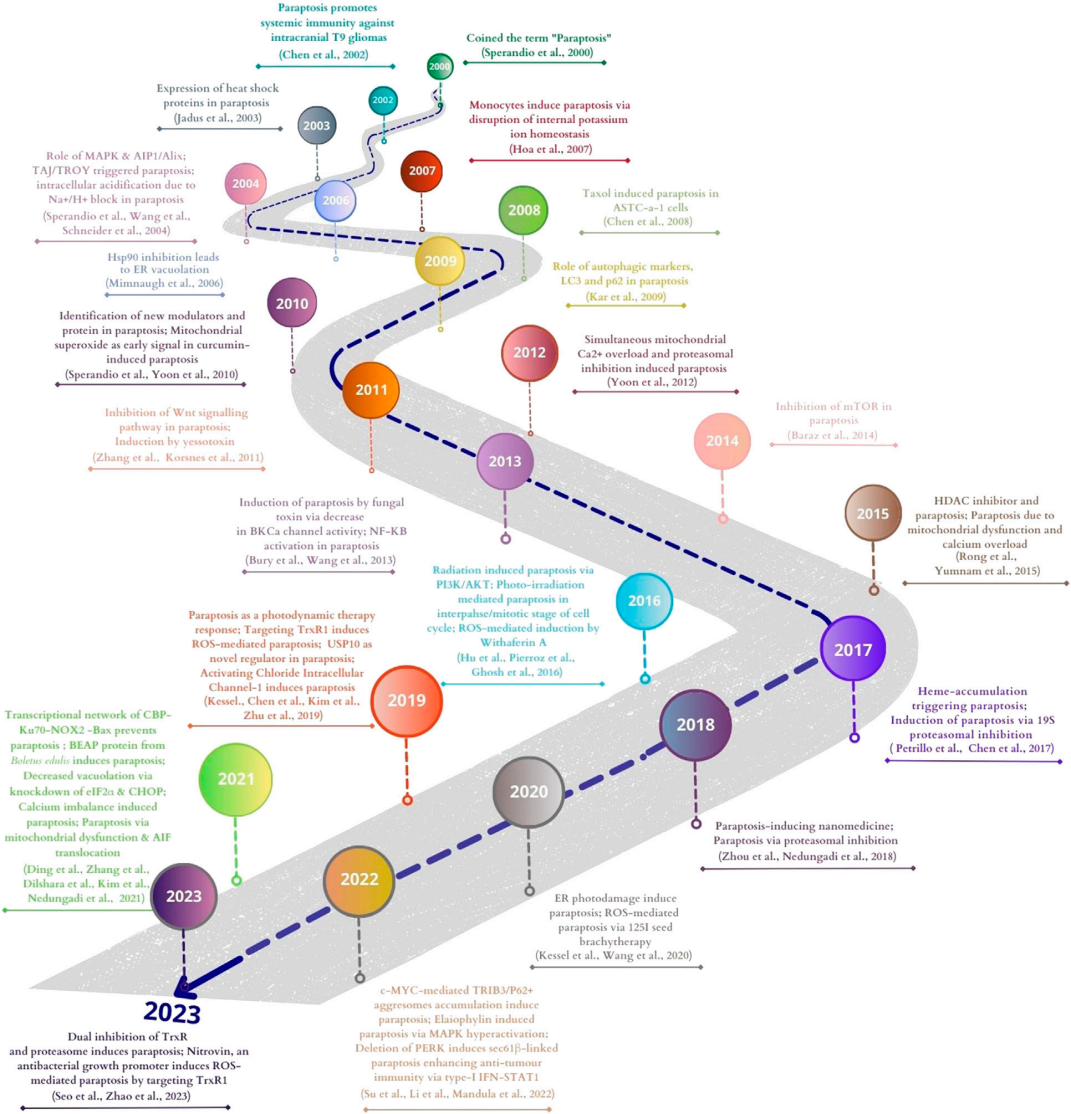
Major milestones of paraptosis research in cancer. This figure was generated using Canva (www.canva.com).

Besides cancer cells gaining resistance to different chemotherapeutic agents, the major concern governing cancer treatment is their ability to evade the most common cell death pathway, apoptosis. Cancer cells are heterogeneous populations that result in different drug sensitivity, resistance to treatment, and induction of different cell death in one population. Hence, we need diverse ways to induce alternate cell death pathways in different cancer cells. Among them, paraptosis is a unique programmed cell death pathway that needs urgent attention for its potential role in cancer treatment. The alternate cell death pathway is a promising field with multiple areas yet to be explored. One reassuring area is studying different targets of cell death like p53, p21, ER stress proteins, mitochondrial proteins, and MAPK pathway proteins that can exert different effects on different cell death pathways and how they potentially induce or suppress them. Different cell cycle regulators’ role during paraptosis is yet to be identified. Till now only a few examples of inhibitors of paraptosis have been described. For comparison, ubiquitous caspase inhibitors such as Z-VAD-fmk and the detection of DNA fragmentation have commonly been used for the characterization and identification of apoptosis. The lack of such inhibitors and markers for the characterization of paraptosis is one of the interesting problems to be solved in paraptosis research. For example, paraptosis can be inhibited by negatively regulating ER stress and/or ROS production but it can also prevent other forms of cell death like apoptosis. Different cell death pathways share biochemical features, like the changes in mitochondria in both apoptosis and paraptosis (Kim et al., 2021). The recent report on the crosstalk between autophagy and paraptosis (Klionsky, 2021; Zhang et al., 2021; Zheng et al., 2022) gives a prospective idea of expanding the research to analyze if different death pathways are connected and whether they have a synergistic or antagonistic effect on each other. Our review recapitulated the different alternative cell death pathways that can be a potential target for cancer treatment, with a prime focus on paraptosis, as they can be concretely activated in cells showing resistance to chemotherapy because of apoptosis inhibition (Blank and Shiloh, 2007). Paraptosis is morphologically characterized by cytoplasmic vacuoles derived from ER and mitochondria, whose functions are altered in malignant cells but not in normal cells. We have also highlighted in this review the various natural compounds that can induce paraptosis, their different modulators, including the markers that are upregulated during the process (e.g., Bip, CHOP, LC3B, p62), the role of profuse ion channels (potassium and calcium channels, BKCa, IP3R), inhibitors like Alix, PEBP-1. The field’s growth is very evident from the literature that shows the manifold application of paraptosis, counting their role in inducing cell death as a result of treatment with nanomedicines (Zhou et al., 2018), metal complexes (Tardito et al., 2009) or photodynamic therapy (Kessel and Reiners, 2020) and their use in combinatorial treatment approaches (Su et al., 2022). Some of the important milestones of paraptosis research are shown in the timeline in Figure 7. The existing reports of *in vivo* studies of paraptosis are very promising, paving the potential future direction of paraptosis research. Many factors that switch on the paraptosis mode in apoptosis-resistant cancer cells are known concerning external agents or stimuli, but some avenues have not yet been worked out, like the micro-RNA and long non-coding RNAs. The epigenetic factors at different levels regulate cell death in eukaryotic cells under pathophysiological conditions like modifying histone proteins and many cytosolic proteins. During therapy, the DNA methylation pattern in different cancers can also give clues to gene expression regulation. Genomic editing tools like CRISPR have opened enormous opportunities for understanding cancer biology and its application in developing new therapeutic targets, diagnosis and prognosis markers. In proteomics, affinity chromatographic enrichment and mass spectrometry have been widely used to identify potential drug-target interactions. In this regard, cellular thermal shift assay (CETSA) is recently being used as a novel technique based on biophysical principles that predict the thermal stabilisation of target proteins *in situ* by ligands that possess the appropriate cellular affinity (Martinez Molina and Nordlund, 2016). For example, Elaiophylin, an autophagy inhibitor, induced paraptosis by hyperactivating the MAPK pathway in multiple cancer cells. Genome-wide CRISPR/Cas9 knockout library screening identified SHP2, an upstream intermediary of the MAPK pathway, as a critical target in Elaiophylin-induced paraptosis. The cellular thermal shift assay (CETSA) and surface plasmon resonance (SPR) assay further confirmed the direct binding between the SHP2 and Elaiophylin (Li et al., 2022a). Thus exploring the changes in paraptosis modulators at the genomics, transcriptomics and proteomics levels in different types of cancer will provide a much better understanding of this unique cell death pathway and its application.
