# Corrigendum: Paraptosis: a unique cell death mode for targeting cancer

**DOI:** 10.3389/fphar.2023.1274076

**Published:** 2023-09-08

**Authors:** Sweata Hanson, Aiswarya Dharan, Jinsha P. V., Sanjay Pal, Bipin G. Nair, Rekha Kar, Nandita Mishra

**Affiliations:** ^1^ School of Biotechnology, Amrita Vishwa Vidyapeetham, Kollam, Kerala, India; ^2^ Department of Cell Systems and Anatomy, UT Health San Antonio, San Antonio, TX, United States

**Keywords:** cancer, paraptosis, programmed cell death, alternative cell death, cancer therapy, apoptosis

In the published article, there was an error in Table 3 as published. The cell line type and the signalling pathway and mechanism attributed to the article by **Garrido-Armas et al. (2018)** was incorrect. The corrected Table 3 appear below.

**TABLE 3:** Paraptosis-inducing compounds against cancer cell lines.

Sl. No:					Compounds	Cell line type								Cell line						Signalling pathway and mechanism						References		
1. Breast																												
i)					Curcumin	Melanocyte								MDA-MB-434S						Inhibition of mitochondrial Na^+^/Ca^2+^ exchanger (mNCX) and proteasome, pERK1/2↑, p-JNKs↑, Alix↓						Yoon et al. 2010 (2012)		
						Epithelial								MDA-MB-231, HS578T														
ii)					Dimethoxy curcumin	Melanocyte								MDA-MB-434S						Proteasomal inhibition and ER stress, pERK1/2↑, p-JNKs ↑, Alix↓						Yoon et al. (2014a)		
						Epithelial								MDA-MB-231, HS578T, MCF-7														
iii)					Celastrol	Melanocyte								MDA-MB-434S						Ca^2+^ overload, proteasomal inhibition via ER stress, pERK1/2↑, p-JNKs ↑, p-p38						Yoon et al. (2014)		
						Epithelial								MCF-7														
iv)					15d-PGJ2	Epithelial								MDA-MB-231						Disruption of sulfhydryl homeostasis, ER stress, pERK1/2↑						Kar et al. (2009)		
v)					Manumycin A	Epithelial								MDA-MB-231, BT-20						ER stress, accumulation of ubiquitinated proteins, p21↑, p27 ↑, PTEN ↑						Singha et al. (2013)		
						Lymphoblast								HCC1937														
vi)					Withaferin A	Epithelial								MDA-MB-231, MCF-7						ER stress, ROS production, Alix↓						Ghosh et al. (2016)		
vii)					Deoxyelephantopin derivative (DETD)	Epithelial								MDA-MB-231						Oxidative and ER stress, p-JNK↑						Shiau et al. (2017)		
viii)					Chalcomoracin	Epithelial								MDA-MB-231						ROS production, PINK1 ↑, Alix ↓, p-ERK↑						Han et al. (2018)		
ix)					6-Shogaol	Epithelial								MDA-MB-231						Proteasomal inhibition, ER stress						Nedungadi, et al. (2018)		
x)					Plumbagin	Epithelial								MDA-MB-231						Disruption of sulfhydryl homeostasis and inhibition of proteasome						Binoy et al. (2019)		
xi)					2′-hydroxy-retrochalcone	Epithelial								MDA-MB-231						Proteasomal dysfunction and ER stress						Nedungadi et al. (2021)		
xii)					Indirubin-3′-monoxime (I3M)	Epithelial								MDA-MB-231						Proteasomal dysfunction and ER stress-mediated Ca^2+^ release.						Dilshara et al. (2021)		
xiii)					Cannabinoids (C6 combination)	Epithelial								MDA-MB-231, MCF-7						ER stress (GRP78 increase)						Schoeman et al. (2020)		
xiv)					Gambogic Acid	Epithelial								MDA-MB-453, MDA-MB-468, MDA-MB-435S						Disruption of thiol proteostasis						Seo et al. (2019)		
						Melanocyte																						
xv)					5,7-dibromo-8-(methoxymethoxy)-2-methylquinoline (HQ-11)	Epithelial								MDA-MB-231, MCF-7						ER stress, proteasomal inhibition, pERK↑						Ma et al. (2022)		
xvi)					Glabridin	Epithelial								MDA-MB-231, MCF-7						ER stress, poly ubiquitinated protein accumulation, proteasome suppression, ROS production, MMP loss						Cui and Cui (2022)		
xvii)					Isoxazole derivative of usnic acid	Epithelial								MDA-MB-231, MCF-7						ER stress, IP3R channel activation						Pyrczak-Felczykowska et al. (2022)		
xviii)					Derivative of pyrazolo[3,4-*h*]quinoline scaffold (YRL1091)	Epithelial								MDA-MB-231, MCF-7						ER stress, accumulation of ubiquitinated proteins, ROS production, ERK↑, JNK↑, Alix↓						Nguyen et al. (2022)		
xix)					Ginger extract	Epithelial								MDA-MB-231						ER stress, mitochondrial dysfunction, AIF translocation and DNA damage						Nedungadi et al. (2021)		
xx)					Disulfiram oxy-derivatives	Epithelial								MCF-7						ER stress, mitochondrial damage, 20S proteasome inhibition and actin depolymerization at later stages						Solovieva et al. (2022)		
2. Brain																												
i)					Curcumin	Glioblastoma								A172						via microRNAs, AKT-Insulin, and p53-BCL2 networks, and AKT protein level reduction was confirmed						Garrido-Armas et al. (2018)		
ii)					Ophiobolin A	Pleomorphicastrocytoid, Neuronal, Fibroblast, Fibroblast) Fibroblast								U373-MG, U251N, U251MG, A172						ER stress, NAC inhibition, decrease of BKCa channel						Bury et al. (2013)		
														T98G														
iii)					Oligomeric Procyanidins	Epithelial								U-87						Extracellular Ca^2+^ influx, pERK1/2↑, p-p38 ↑						(Zhang et al., 2010)		
iv)					Paclitaxel	Epithelial								U-87						CHX has no effect, MEK, p38 and JNK pathways are not involved						Sun et al. (2010)		
v)					Yessotoxin	Muscle cells from intracranial tumor								BC3H1						ER and mitochondrial swelling, p-JNK↑						Korsnes et al. (2011)		
vi)					1-Desulfo Yessotoxin	Muscle cells from intracranial tumor								BC3H1						ER and mitochondrial swelling, p-p38↑						Korsnes et al. (2013)		
vii)					Xanthohumol	Epithelial								SH-SY5Y						ER stress and LC3B upregulation, p38 ↑						Mi et al. (2017)		
3. Blood																												
i)					Honokiol	Lymphoblast								K562						ROS generation ROS generation, ER stress, LC3 upregulation, mTOR and MAPK activated						Liu et al. (2021), Wang et al. (2013)		
						Promyelocyte								NB4														
ii)					Xanthohumol	Promyeloblast								HL-60						ER stress and LC3B upregulation, p38 ↑						Mi et al. (2017)		
iii)					Iturin lipopeptide	Lymphoblast								K562						LC3B and p62 upregulation						Zhao et al. (2019)		
iv)					Brassinin	Lymphoblast								K562						ROS production, mitochondrial damage, ER stress, and activation of MAPK						Yang et al. (2023)		
						Lymphoblast-like								KBM5, LAMA84, and KCL22														
4. Cervical																												
i)					Celastrol	Epithelial								HeLa						Proteasome inhibition, Mitochondrial Ca^2+^ overload, pERK1/2↑, p-JNKs ↑, p-p38 ↑						Wang et al. (2012)		
ii)					Cyclosporin A	Epithelial								HeLa, SiHa						LC3 upregulation, Cyclophilin B↓, Alix↓						Ram and Ramakrishna (2014)		
iii)					8-p-Hydroxybenzoyl tovarol	Epithelial								HeLa						Bip, CHOP, IRE1α and XBP1 upregulation						Zhang et al. (2015)		
iv)					Seleno-DL-Cystine	Epithelial								HeLa						Bip and CHOP polyubiquitination upregulation, ROS generation						Wallenberg et al. (2014)		
v)					Paclitaxel	Epithelial								HeLa						CHX has no effect, MEK, p38 and JNK are not involved						Sun et al. (2010)		
vi)					Wheat germ Agglutinin	Epithelial								HeLa, SiHa, CaSKi						Autophagy-linked FYVE (Alfy) protein inhibition, ER stress, LC3B upregulation						Tsai et al. (2017)		
vii)					2′-hydroxy-retrochalcone	Epithelial								HeLa						Proteasomal dysfunction, ER stress, LC3 upregulation						Nedungadi et al. (2021)		
5. Thyroid																												
i)					Tunicamycin	Epithelial								8505C, CAL62, FRO cell lines						Bip, CHOP, p-PERK and IRE1 upregulation						Kim et al. (2014)		
6. Liver																												
i)					Hesperidin	Epithelial								HepG2						Mitochondrial dysfunction and Ca^2+^ overload, p-ERK↑						Yumnam et al., 2016)		
ii)					Cis-Nerolidol	Epithelial								HepG2/C3 A						ER stress, increased activity of cytochrome P450 enzymes						Biazi et al. (2017)		
iii)					Gambogic Acid	Epithelial; diffusely spreading cells								SNU-449						Proteasomal inhibition and ER stress, ROS independent- mitochondrial depolarization						Seo et al. (2019)		
7. Colon																												
i)					Curcumin	Epithelial								HCT116						Proteasome inhibition ROS, Mitochondrial Ca^2+^ overload, LC3 upregulation, pERK1/2↑, p-JNKs↑, Alix↓						Lee et al. (2015)		
ii)					Celastrol	Epithelial								DLD-1, RKO						Proteasome inhibition, Mitochondrial Ca^2+^ overload, pERK1/2↑, p-JNKs ↑, p-p38 ↑						Yoon et al. (2014)		
iii)					15d-PGJ2	Epithelial								HCT116						Disruption of sulfhydryl homeostasis LC3 upregulation, pERK1/2↑						Kar et al. (2009)		
iv)					Ginsenoside Rh2	Epithelial								HCT116, SW480						p53 activation, activation of death by antioxidants						Li et al. (2011), Wan et al. (2018)		
v)					Protopanaxadiol	Epithelial								HCT116, SW480						Death acceleration by inhibiting ROS generation, NF-κB activated						Wang et al. (2013)		
vi)					ɣ-Tocotrienol	Epithelial								SW620 and HCT-8						Wnt signals↓ (β-catenin, cyclin D, c-Jun)						Zhang et al. (2011) (2013)		
					δ-Tocotrienol	Epithelial								SW620						Wnt signals↓ (β-catenin, cyclin D, c-Jun)								
vii)					Iturin A-like lipopeptides	Epithelial								Caco-2						ER stress, ROS generation, Ca^2+^ ↑						Zhao et al. (2019)		
viii)					Loperamide	Epithelial								DLD-1, SW-480, SW-620, HCT116						ER stress, Ca^2+^ imbalance and CHOP↑						Kim et al. (2019)		
ix)					Purified resin glycoside fraction (Pharbitidis Semen)	Epithelial								HT-29 and HCT-116						Chloride intracellular channel-1 activation and intracellular Cl^−^↑, MAPK activation						Zhu et al. (2019)		
8. Prostate																												
i)					Curcumin	Epithelial								PC-3M						Proteasome inhibition ROS, Mitochondrial Ca^2+^ overload, LC3 upregulation, pERK1/2↑, p-JNKs↑, Alix↓						Lee et al. (2015)		
ii)					15d-PGJ2	Epithelial								DU145						Disruption of sulfhydryl homeostasis LC3 upregulation, pERK1/2↑						Kar et al. (2009)		
iii)					Benzo[a]quinolizidine analogs	Epithelial								PC3						ER stress and LC3B upregulation						Zheng et al. 2016)		
iv)					Chalcomoracin	Epithelial								LNCaP, PC-3						ROS generation, ER stress, PINK1 ↑, Alix ↓, p-ERK↑						Han et al, 2018)		
v)					δ-Tocotrienol	Epithelial								CRPC cells—DU145, PC-3						ER stress, LC3 and p62 upregulation, p-JNK ↑, p-p38 ↑						Fontana et al. (2020)		
9. Ovarian																												
i)					Morusin	Epithelial								A2780, HO-8910, SKOV3						Ca^2+^ overload, ROS generation and loss of mitochondrial membrane potential						Xue et al. (2018)		
ii)					Elaiophylin	Epithelial								SKOV3, OVCAR8, UWB1.289, SW626						ER stress, SHP2/SOS1/MAPK↑						Li et al. (2022)		
10. Lung																												
(i)					Cyclosporin A	Epithelial								A549						LC3 upregulation, Cyclophilin B↓, Alix↓						Ram and Ramakrishna (2014)		
ii)					Paclitaxel	Epithelial								A549						CHX has no effect, MEK, p38 and JNK are not involved						Guo et al. (2010)		
						Epithelial								ASTC-a-1														
iii)					6-Shogaol	Epithelial								A549						Proteasome inhibition, ER stress, ROS generation, LC3 upregulation						Nedungadi et al. (2018)		
iv)					Hinokitiol copper complex	Epithelial								A549						Proteasome inhibition, ER stress						Chen et al. (2017)		
v)					Chalcomoracin	Epithelial								H460						ER stress, MAPK activation						Han et al. (2018)		
						Epithelial								A549														
						Adenocyte								PC-9														
vi)					Paris Saponin II (PSII)	Epithelial								NCI-H460						ER stress, JNK pathway activation						Man et al. (2020)		
						Epithelial								NCI-H520														
vii)					Prenylated bibenzyls (*Radula constricta*)	Epithelial								A549, NCI-H1299						ROS elevation and loss in mitochondrial membrane potential						Zhang et al. (2019)		
viii)					Gambogic Acid	Epithelial								NCI-H460						Proteasomal inhibition and ER stress, ROS independent- mitochondrial depolarization						Seo et al. (2019)		
ix)					Epimedokoreanin B	Epithelial								A549, NCL-H292						ER stress, autophagosome accumulation, ROS production, loss of MMP, UPR signaling						Zheng et al. (2022)		
x)					DHW-221	Epithelial								A549						ER stress, PI3K/mTOR inhibition, MAPK activation						Liu et al. (2022)		
xi)					Ginger extract	Epithelial								A549						ER stress, mitochondrial dysfunction, AIF translocation and DNA damage						Nedungadi et al. (2021)		
11. Skin																												
i)					Cyclosporin A	Keratinocyte								HaCaT						LC3 upregulation, Cyclophilin B↓, Alix↓						(Ram and Ramakrishna (2014)		
ii)					δ-tocotrienol	Epithelial								A375						Ca^2+^ overload and ROS generation, MAPK activation						Raimondi et al. (2021)		
12. Bone																												
i)					Cyclosporin A	Epithelial								U2OS, Saos-2						LC3 upregulation, Cyclophilin B↓, Alix↓						Ram and Ramakrishna (2014)		
13. Kidney and Bladder																												
i)					Jolkinolide B	Epithelial								T24, UM-UC-3, T24/CDDP						ROS-mediated ER stress, MAPK and ERK activation						Sang et al. (2021)		
14. Oral																												
i)					Isorhamnetin (3′-Methoxy-3,4′,5,7-tetrahydroxyflavone)	Epithelial								HSC-3, HSC-4, PE/CA-PJ15						↑ROS generation, ERK/MAPK						Chen et al. (2021)		
15. Pancreas																												
i)					Gambogic Acid	Epithelial								BxPC-3						Proteasomal inhibition and ER stress, ROS- independent mitochondrial depolarization						Seo et al. (2019)		
16. Stomach																												
i)					Gambogic Acid	Epithelial								SNU-668 (gastric cancer)						Proteasomal inhibition and ER stress, ROS independent- mitochondrial depolarization						Seo et al. 2019)		

The authors apologize for this error and state that this does not change the scientific conclusions of the article in any way. The original article has been updated.

